# Divergence in evolutionary potential of life history traits among wild populations is predicted by differences in climatic conditions

**DOI:** 10.1093/evlett/qrad067

**Published:** 2024-02-01

**Authors:** Stéphane Chantepie, Anne Charmantier, Boris Delahaie, Frank Adriaensen, Erik Matthysen, Marcel E Visser, Elena Álvarez, Emilio Barba, Markku Orell, Ben Sheldon, Elena Ivankina, Anvar Kerimov, Sébastien Lavergne, Céline Teplitsky

**Affiliations:** Université Grenoble Alpes, CNRS, Univ. Savoie Mont Blanc, CNRS, LECA, Laboratoire d’Écologie Alpine, Grenoble, France; Institut de Systématique, Évolution, Biodiversité, École Pratique des Hautes Études, Paris Sciences et Lettres, Muséum National d’Histoire Naturelle, CNRS, Sorbonne Université, Université des Antilles, Paris, France; INRAE, ONF, BIOFORA, 45075, Orléans, France; CEFE, Univ Montpellier, CNRS, EPHE, IRD, Montpellier, France; CIRAD, UMR DIADE, Montpellier 34398, France; UMR DIADE, Université de Montpellier, CIRAD, IRD, Montpellier, France; Evolutionary Ecology Group, Department of Biology, University of Antwerp, Wilrijk, Belgium; Evolutionary Ecology Group, Department of Biology, University of Antwerp, Wilrijk, Belgium; Department of Animal Ecology, Netherlands Institute of Ecology, Wageningen, The Netherlands; Cavanilles Institute of Biodiversity and Evolutionary Biology, Department of Microbiology and Ecology, University of Valencia, Paterna, Spain; Cavanilles Institute of Biodiversity and Evolutionary Biology, Department of Microbiology and Ecology, University of Valencia, Paterna, Spain; Ecology and Genetics Research Unit, Faculty of Science, University of Oulu, Oulu, Finland; Edward Grey Institute, Department of Biology, University of Oxford, Oxford, United Kingdom; Zvenigorod Biological Station of Lomonosov Moscow State University, Moscow, Russia; Department of Vertebrate Zoology, Faculty of Biology, Lomonosov Moscow State University, Moscow, Russia; Université Grenoble Alpes, CNRS, Univ. Savoie Mont Blanc, CNRS, LECA, Laboratoire d’Écologie Alpine, Grenoble, France; CEFE, Univ Montpellier, CNRS, EPHE, IRD, Montpellier, France

**Keywords:** G-matrix, wild populations, climatic niche, great tits

## Abstract

Short-term adaptive evolution represents one of the primary mechanisms allowing species to persist in the face of global change. Predicting the adaptive response at the species level requires reliable estimates of the evolutionary potential of traits involved in adaptive responses, as well as understanding how evolutionary potential varies across a species’ range. Theory suggests that spatial variation in the fitness landscape due to environmental variation will directly impact the evolutionary potential of traits. However, empirical evidence on the link between environmental variation and evolutionary potential across a species range in the wild is lacking. In this study, we estimate multivariate evolutionary potential (via the genetic variance–covariance matrix, or G-matrix) for six morphological and life history traits in 10 wild populations of great tits (*Parus major*) distributed across Europe. The G-matrix significantly varies in size, shape, and orientation across populations for both types of traits. For life history traits, the differences in G-matrix are larger when populations are more distant in their climatic niche. This suggests that local climates contribute to shaping the evolutionary potential of phenotypic traits that are strongly related to fitness. However, we found no difference in the overall evolutionary potential (i.e., G-matrix size) between populations closer to the core or the edge of the distribution area. This large-scale comparison of G-matrices across wild populations emphasizes that integrating variation in multivariate evolutionary potential is important to understand and predict species’ adaptive responses to new selective pressures.

## Introduction

Along with phenotypic plasticity and range shifts toward suitable environments, evolution represents a key mechanism for natural populations to persist under consistently adverse environmental conditions (Chevin et al., 2010). The rate and direction of evolutionary response critically depend on both natural selection and available standing genetic variance (Walsh & Lynch, 2018). The joint response to selection of multiple traits can be facilitated or constrained by the genetic association among traits (Agrawal & Stinchcombe, 2009; Bolstad et al., 2014; Schluter, 1996; Stinchcombe & Kirkpatrick, 2012; Teplitsky et al., 2014). Under this framework, the matrix of additive genetic variances and covariances (G-matrix), which summarizes the multivariate standing genetic variance, plays a fundamental role in predicting the response to selection as the adaptive response across a single generation is given by the product of the G-matrix and the selection gradient (Lande, 1979). The extrapolation of the adaptive response to selection over multiple generations or across space thus largely depends on the constancy of the G-matrix in time and space. The stability of the G-matrix is a paramount assumption, prevalent in quantitative genetic models (Duputié et al., 2012).

The structure of G-matrices results from the interactive effects of selection, mutation, drift, and migration processes (Steppan et al., 2002). Since these processes, which jointly affect allele frequencies across populations, can change both in space and time, theory predicts that G-matrices should also vary (Chantepie & Chevin, 2020). Spatiotemporal changes in the G-matrix can be best interpreted in geometrical terms (Jones et al., 2004) such as their variation in size (overall evolutionary potential), orientation (direction of fastest evolution), and shape (constraint resulting from genetic correlation). In finite populations, the shape of the G-matrix is a compromise between the shape and orientation of the matrix of selective gradients and the matrix of pleiotropic mutations (Jones et al., 2007, 2014; Revell, 2007). At mutation–selection equilibrium, when drift is high, the G-matrix tends to orient toward the direction of the mutational matrix (Chantepie & Chevin, 2020). Differences in these forces among populations will contribute to differentiation among G-matrices, while gene flow is expected to homogenize allele frequencies across populations hence reducing the spatial variation in G-matrices (Guillaume & Whitlock, 2007; Lande, 1992). Finally, drift may also lead to random divergence of G-matrices among populations (Phillips et al., 2001). Experimental studies conducted over the last decades have demonstrated that each of the above-mentioned evolutionary forces can significantly affect the geometry of the G-matrix (examples for drift: Phillips et al., 2001, selection: Cano et al., 2004; Careau et al., 2015; Doroszuk et al., 2008; Shaw et al., 1995, dispersal: Nosil et al., 2006). In addition to evolutionary forces, gene expression depending on the environment (**G** × **E**) can generate variation in the G-matrix independently of changes in allele frequencies (Wood & Brodie, 2015).

Although experimental studies are valuable for understanding how evolutionary forces can shape the G-matrix and testing theoretical hypotheses, the extrapolation of laboratory results to wild populations living under natural conditions remains challenging (Kruuk et al., 2008). In particular, the difficulty to measure precisely each of these evolutionary forces as well as their interactions makes it impossible to predict theoretically the variation in G-matrices in the wild (Arnold et al., 2008; Roff, 2000). Investigating this variation among populations can provide inference on forces driving spatial differences but also, using space for time substitution, it can contribute to improve predictions of temporal changes in G-matrices. Addressing the spatial variation in the G-matrix requires thus not only to characterize the extent to which the G-matrix varies among populations but also the spatial determinants (e.g., geographic distance, environmental conditions) that have shaped this variation. So far, the literature relative to G-matrix variation among populations has provided contrasting results with evidence for both stability (Ashman, 2003; Brodie, 1993; Delahaie et al., 2017; Hangartner et al., 2020; Henry & Stinchcombe, 2023; Puentes et al., 2016; Sniegula et al., 2018) and variation (Berger et al., 2013; Eroukhmanoff & Svensson, 2011; Paccard et al., 2016; Roff et al., 2004; Teplitsky et al., 2011) of the G-matrix across various spatial gradients in plant and animal populations. The extent to which differing environmental conditions contribute to explain G-matrix differentiation is still rarely explicitly investigated, e.g., by correlating G-matrix geometry to environmental variables. Changes in environmental conditions are expected to have a greater impact on the selection regime for life history traits than for morphological traits (e.g., Lundblad & Conway, 2020 in birds) because life history traits are directly affected by the environment. We thus predict that variation among the G-matrix for life history traits should be more strongly correlated to environmental variation than variation among G-matrices for morphological traits (Arnold et al., 2008).

Linking variation in G-matrices among populations with environmental variation requires to define relevant environmental factors describing global differences in selective pressures across a species distribution range. A meta-analysis across plants and animals highlighted the relation between climatic conditions (temperature and precipitation) and spatial variation in selection coefficients (Siepielski et al., 2017, 2018). The set of climatic conditions where a species’ population can have a positive growth rate (termed climatic niche hereafter) thus appears as an appropriate integrative description of the environmental space that might shape the selection regime of traits and the among-population variation in G-matrices. In line with this, evolvability of single traits in bird populations tends to be higher for intermediate levels of climatic favorability (Martínez-Padilla et al., 2017). More generally, the reduction in effective sample size due to harsh environmental conditions, such as low-climatic favorability experienced by populations at the niche border, could result in a global reduction of additive genetic variance (Polechová & Barton, 2015) and has been advocated as the primary mechanism for explaining species distribution boundaries (Holt et al., 2008). Thanks to the development of long-term monitoring of wild populations (Culina et al., 2021) with replicated populations for some model species in ecology, it is now possible to compare G-matrices across populations ranging from the center to the edge of the species’ climatic niche.

Estimation of the G-matrix in wild populations requires datasets with measures of phenotypic traits and associated pedigree over several decades. In this study, we took advantage of 10 exceptional long-term datasets on great tits (*Parus major*) populations monitored in the wild across Europe from Spain to Finland and Russia to estimate and compare G-matrices among contrasted environmental conditions. The overall genetic differentiation (*F*_st_) between European populations of great tits has been shown to be low (Laine et al., 2016; Lemoine et al., 2016; Spurgin et al., 2019), suggesting a limited impact of drift and founder effects on the variation among G-matrices compared to environmental drivers. G-matrices were inferred separately for morphological and life history traits in each population using “animal models” under a Bayesian framework. Based on climatic data available at large spatial scales and high resolution, we constructed the climatic niche of great tits in their distribution range in order to (a) investigate the association between the relative variation in G-matrices among populations and their distances within the climatic niche for both trait categories and (b) test whether populations closer to the niche center showed more genetic variation than populations at the edge of the niche.

## Methods

### Great tit datasets

**Figure 1.**
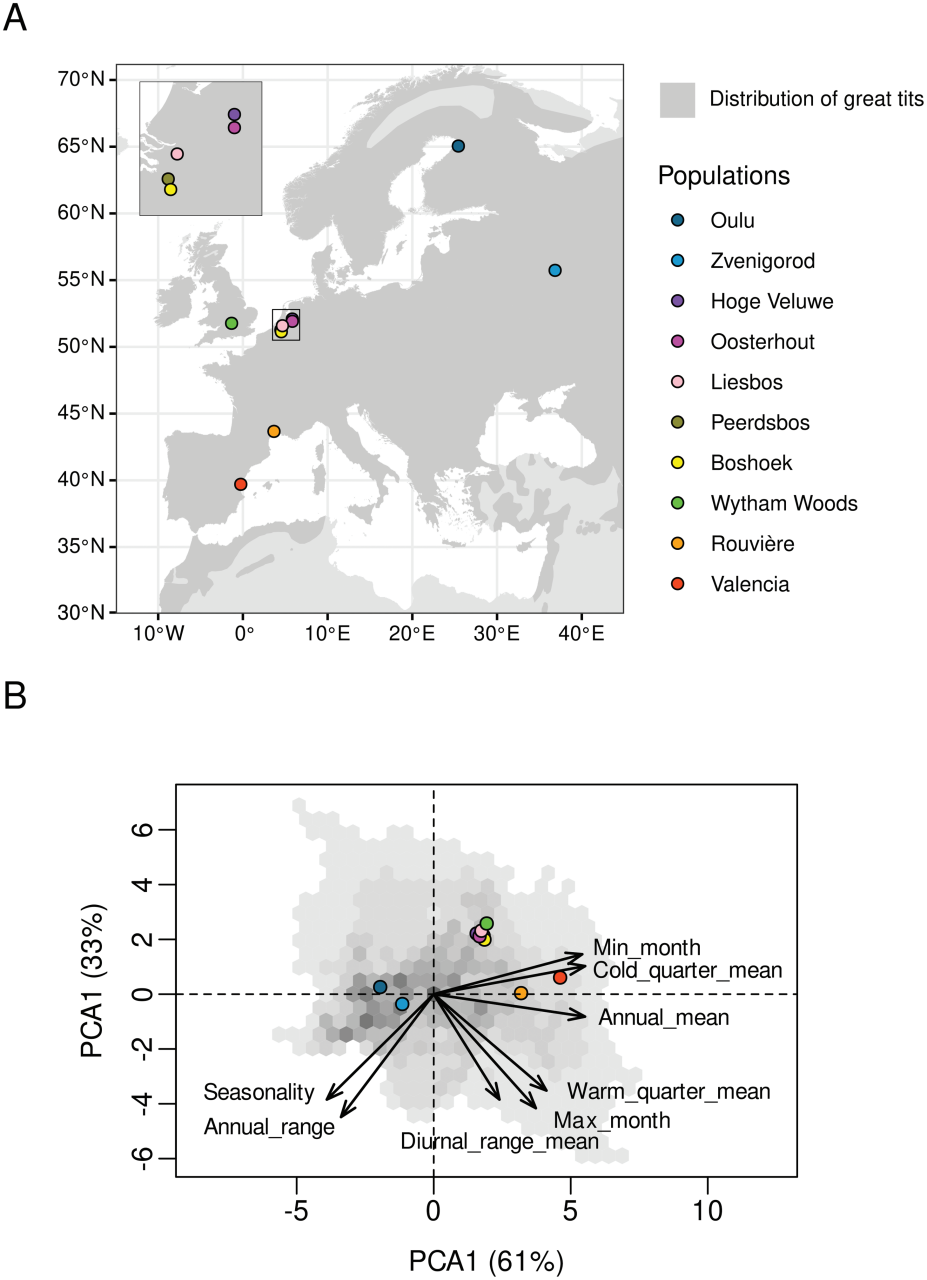
Location of populations within space and climatic niche. (A) Geographical distribution of great tits with the location of the 10 focal populations. (B) Climatic niche of great tits. Results of the principal component analysis realized on the climatic variables (array) used at a resolution of 5 km^2^. Points correspond to the predicted position of the 10 populations within the climatic niche. The gray gradient represents the frequency of the climatic conditions across the distribution and ranges from 1 (lightest gray) to 10^4^ cells (darkest gray). The full names of the temperature variables are available in *Methods* section.

The meta-dataset used in this study represents a collection of 10 populations of great tits monitored in the wild at different locations across Europe (Figure 1). These populations have been monitored for at least 20 years with a maximum of 59 years (Supporting Information 1). In all these populations, birds were banded either as chicks or as breeding parents at their nest with a unique identifying metal ring. This identification was used to build a trans-generational social pedigree in each population (Supporting Information 2, Supplementary Tables S8 and S9).

Focal phenotypic traits consisted of both morphological and life history features. Three morphological measurements were taken on both male and female breeders: tarsus length (in mm), flattened wing length (in mm), and body mass (in g). For the two Belgian populations, mean tarsus length was slightly smaller than in the other populations due to a different measurement method (pers. comm., Matthysen) (Supporting Information 1, Supplementary Table S1). Similarly, for the Dutch populations, the measurement method led to smaller wing lengths than in other populations. However, additive differences in measures do not impact variance estimates (Hansen et al., 2011). Unlike for morphological traits, only females were considered for the three life history traits analyzed, namely laying date (date of first egg laid in the nest, day 1 = Jan 1st), clutch size (number of eggs), and fledging success (number of chicks leaving the nest). Although great tits can produce a second clutch in some years or may replace a first clutch after a breeding failure, only the first clutch of the year was considered for each female. Fledging success was zero truncated because breeding failure was not recorded similarly in all populations (failures to lay any egg vs. failures following the first egg laid) and can result from many different causes (e.g., climatic event, predation, and experimental effect).

### Estimation of G-matrix

G-matrices were estimated for each population by using multivariate animal models (Kruuk, 2004; Walsh & Lynch, 2018). Distinct animal models were fitted for morphological and life history traits. Multivariate animal models represent a particular form of multivariate mixed models that allow the partitioning of phenotypic variance into its genetic and nongenetic components. The G-matrix was estimated by fitting a random effect based on the coefficient of coancestry among pairwise individuals, inferred from pedigree data (Kruuk, 2004). The nongenetic components in our animal models included a permanent environmental effect (PE-matrix) that accounted for the nonindependence of repeated measurements of the same individual, a year effect (Y-matrix) for nonindependence of values with respect to years and a residual variance (R-matrix). We also fitted the observer ID (Obs-matrix) as a random effect on wing and tarsus length when information was available. For all traits, we fitted age as a fixed effect (as a quadratic continuous function or age classes according to populations). For morphological traits, additional fixed effects for sex, sex by age interaction, and date of measurement (as cubic continuous or time periods) were fitted. The fixed and random parameters were adapted to each dataset to account for differences in data collection (Supporting Information 2, Supplementary Table S10). We dealt with missing fixed quantitative parameters with two strategies using (a) the average data augmentation or (b) the missing data deletion. Although both strategies may bias the parameter estimates (Nakagawa, 2015), no difference was found between the G-matrix estimates depending on the strategy used (not shown). Only the results with the average data augmentation are presented here.

We ran the animal mixed models in a Bayesian framework using the MCMCglmm R-package (Hadfield, 2010). The posterior distribution of each parameter estimates corresponded to $10^{3}$ independent samples along the Monte Carlo chain. For all animal models, we started with a burn-in phase of $2.10^{5}$ iterations followed by an estimation phase of $10^{6}$ iterations where parameter estimates were sampled every $10^{3}$ steps. The convergence of the models was assessed by visually checking the posterior estimates and ensuring that autocorrelation of all parameter estimates was lower than 0.1. When necessary, both the number of iterations and sample steps were increased to meet the autocorrelation threshold requirement. To facilitate convergence, the animal models were run using slightly informative inverse Wishart prior distributions for each component of the variance. For each population, the scale parameter prior was defined as a diagonal matrix containing the phenotypic variance of traits divided by the number of random factor in the model on the diagonal (Hadfield, 2010). Finally, the degree of belief for priors was set equal to the number of traits.

### G-matrix standardization

We used the same traits in all populations, but these traits differ widely in terms of scales and units (e.g., gram vs. mm), leading to a risk that larger measurements could disproportionately affect potential differences among G-matrices (Hansen et al., 2011). When the values of a trait are several orders of magnitude higher than in other traits, almost all the variation in G-matrix is explained by this trait. We thus standardized the G-matrices posterior estimates before running comparisons among populations.

Standardization is classically done relatively to the trait overall mean or to the phenotypic variance. However, standardization to the phenotypic mean, considered as a natural measure of evolvability, could not be used here because no natural mean exists for variables expressed on an interval scale such as laying date and may not be appropriate for clutch size (Hansen & Houle, 2008; Pélabon et al., 2020). Furthermore, in natural populations, standardization to the phenotypic variance may lead to strongly biased results due to the correlation between additive genetic variance and environmental variance (Hansen et al., 2011).

Variance ratios of similar traits (e.g., phenotypes and breeding values) are unitless, scale invariant, and biologically interpretable (as the percentage of change between the two trait variances) (Le Maître & Mitteroecker, 2019). In this study, G-matrices were standardized to the interpopulation mean of traits additive genetic variance $\left(\bar{Va}\right)$. Within traits, additive genetic variances of all populations are divided by the same constant value (mean traits additive genetic variance), so that the relative variation of these genetic variances among populations is not affected by standardization. Similarly, genetic covariances were divided by the square root of the product of mean traits additive genetic variances to preserve the genetic correlation among traits. Overall, this standardization is focusing on preserving the relative variation in variance and covariance of traits among populations (see Supporting Information 3 for details) in order to adequately describe the variations among G-matrices but was not intended to give insights on the difference in absolute evolvability among traits. To estimate $\bar{Va}$, we first drew a thousand multivariate normal estimates for each posterior sample of all G-matrix, and then calculated the variance of all merged estimates. The interpopulation mean additive genetic variance for each trait can be interpreted as the trait-specific genetic variance of species, assuming that the populations are panmictic. A variance ratio above one thus means that the population harbors more genetic variance than average for this trait. We provided unstandardized G-matrix, P-matrix, and heritability for all traits in Supporting Information 4 (Supplementary Tables S11–S17).

### Null model

To ensure that our results do not arise solely from sampling effects and uncertainty in the estimation of G-matrices, a null model was developed following the general framework of Morrissey et al. (2019). For each population, the breeding values of founders were drawn from a multivariate distribution with zero mean and a variance–covariance matrix equal to the G-matrix estimated for this population with the animal model. These breeding values were then randomly shuffled among the founders from the different populations. This random attribution of breeding values simulates a unique G-matrix for all populations, from which population genetic values are sampled. Based on these random breeding values for founders and each population’s pedigree, we estimated the breeding values of all remaining individuals following the infinitesimal assumption. The breeding values of an individual were drawn from a multivariate normal distribution with a vector of means equal to the mean breeding values of its parents and a deviation equal to the segregation variance (Walsh & Lynch, 2018). For an individual, the segregation variance was estimated as half the product of the randomized G-matrix of their population (estimated on the new population’s founders) and one minus the average inbreeding coefficient of its parents (Walsh & Lynch, 2018). For each population, the phenotypes were then recomposed by adding to the simulated breeding values the random and fixed effects estimated by the animal model run on the wild dataset. The fixed effects were estimated as the product of the fixed-effect design matrix and the vector of fixed effects drawn from the posterior sample of the animal model for each population. Random effects were drawn from a multivariate distribution with a mean of zero and their respective variance–covariance matrix resulting from the animal models (i.e., PE-matrix, Y-matrix, Obs-matrix, and R-matrix). Original populations estimates for random effects other than additive genetic variance were kept because the amount of heritability can affect the power of the analysis (Morrissey et al., 2007) and effects such as year effect or permanent environment are environmental and thus population specific.

This sequence of generating phenotypes was repeated for each posterior sample of animal models and resulted in 1,000 null datasets for each population. The G-matrix of null datasets was estimated with the same animal models used for the corresponding wild population dataset. To reduce computational burden, we sampled 200 posterior samples for each null animal model (Morrissey et al., 2019) to compute a mean null distribution G-matrix for each population.

### Climatic data and niche modeling

The climatic niche of great tits was estimated using known species occurrences and climatic data available at a large geographical scale. We used the great tit distribution map from Birdlife International and Nature Serve (Birdlife International and Nature Serve, 2018), which includes all subspecies. In this studies, we only considered European subspecies of the great tit and restricted geographic distribution to the West Palearctic region (Kvist et al., 1999). We extracted temperature data from WorldClim2 Global Climate GIS data at a 2.5 min resolution (∼5 km) over the species distribution (Fick & Hijmans, 2017). The WorldClim2 contained climate data that were monthly averaged for the 1970–2000 period, a scale relevant for our study as we were interested in large-scale climate variation (rather than weather anomalies) and since founders of the populations (i.e., individuals whose parent is unknown) could enter the datasets any year during the monitoring (either as immigrants or chicks born outside of nest boxes). The temperature variables included to build the climatic niche were: the annual mean temperature, mean diurnal temperature range, temperature seasonality, maximum temperature of warmest month, minimum temperature of coldest month, temperature annual range, mean temperature of warmest quarter, and mean temperature of coldest quarter (all temperatures in degrees Celsius).

To estimate the niche space, a principal component analysis (PCA) was performed on these eight climatic variables over all cells included in the distribution of great tits (Figure 1A). We assessed the position of each population within the bioclimatic niche by (a) extracting the local Worldclim2 climatic conditions at their respective geographical coordinates and (b) projecting these local conditions on the two first PCA axes (Figure 1B). The niche distances among populations were estimated as the Euclidean distance among the projected population positions within the first two axes PCA space. As the niche distance and geographic distance were highly correlated, only the former was used for analyses. We considered the origin of the PCA space as the center of the bioclimatic niche and estimated the Euclidean distance between the center of the niche and the position of the projected populations.

### Statistical differences in G-matrices among populations

#### Genetic covariance tensor analysis

Differences in genetic covariance structure across populations were tested using genetic covariance tensors (Hine et al., 2009). Instead of performing pairwise comparisons between matrices, the tensor method investigates the changes in the structure of G-matrices along a gradient. The tensor approach describes the variation among G-matrices based on successive linear transformations and identifies independent combinations of traits displaying a change in genetic variance. Detailed descriptions of the method can be found in Aguirre et al. (2014), Walter et al. (2018), and Supporting Information 5.

Succinctly, a genetic covariance tensor was built from the variances and covariances between the elements of the G-matrices. An eigen-decomposition of this covariance tensor was performed to assess the independent axes of variation among the G-matrices. The eigenvectors of this decomposition, called eigentensors, represent the directions which maximize the variance among the original G-matrices. The magnitude of the variation among the G-matrices along a particular eigentensor was determined by the corresponding eigenvalues. The coordinates of each G-matrix in the space defined by the eigentensors describe how much the populations differ along a given eigentensor. In other words, when two G-matrices are close with respect to a particular tensor, then these matrices are described in the same way by the eigentensor. The eigentensor itself can be eigen-decomposed to eigenvectors that assess which independent linear combinations of traits have contributed to the change in G-matrix structure along this tensor. The corresponding eigenvalue of the eigenvectors describes the magnitude of the contribution of trait in these changes. We applied this method to the posterior estimates of the animal models (Aguirre et al., 2014) and compared the results to our null model.

The distances among populations were estimated as the Euclidean distance among coordinates in the space defined by the two first tensors (hereafter “tensor distance”). To evaluate the extent to which climatic conditions are driving the differentiation among G-matrices, first, we tested the relation between the tensor distances estimated with posterior mode of coordinates and niche distances using a Mantel test. Second, to account for the uncertainty in coordinates estimates on the tensors, we also estimated 1,000 tensor distances matrix using the posterior distribution of coordinates. To remove the dependence among the tensor distances and allow for estimating correlation with niche distance, we shuffled each element among the 1,000 distance matrices. Finally, we performed linear regressions with randomized tensor distance as the dependent variable and the niche distance as the explanatory variable for all the tensor distance matrices and tested significance of the slope using the 95% credible interval (CI).

#### Orientation, shape, and size of G-matrices

We assessed the overall structure of each G-matrix through eigenanalyses. The major eigenvector of a G-matrix called *g*_max_ is oriented in the direction of maximum genetic variance in the population in the standardized space of traits. The ratio between the eigenvalue of *g*_max_ and the sum of all the eigenvalues provides information about the eccentricity of the G-matrix, or how much the response to selection is favored in the direction of *g*_max_.

The angle between two populations refers to the variation in their direction of fastest evolution in the standardized space of traits. We estimated the critical angles between *g*_max_ of two G-matrices using the method of Krzanowski (1979). We examined the statistical support for two populations having different *g*_max_ by comparing the intrapopulation variation of *g*_max_ (drawn from the posterior distribution) with the interpopulation variation *g*_max_ (Ovaskainen et al., 2008) (Supporting Information 6). To evaluate whether the direction of fastest evolution diverged according to the difference in climatic conditions, the angles between *g*_max_ were compared against niche distance using Mantel test and linear regressions (see details above).

To test for the hypothesis of a decrease in the total relative genetic variance or total relative size from the center to the edge of the niche, we used the volume of each G-matrix as a proxy of the total genetic variance. For each G-matrix, we estimated the posterior distribution of volume as the sum of the eigenvalues for all independent posterior samples of the G-matrices. Then, we performed a linear regression modeling the posterior volume distribution as a function of the distance of the population to the center of the niche (significance of the slope tested using the 95% CI).

## Results

### Climatic niche and phenotype divergence across populations

The 10 populations of great tits used in this study were distributed over a wide geographic area (distance between populations ranged from 2 to 3,300 km) and were found in contrasting environments (Figure 1B). The first two axes of the PCA accounted for 61% and 33% of the variance in bioclimatic data, respectively. The first axis of the PCA explained broad variations in temperature within the distribution range of great tits and the second axis mostly described environmental variability (Figure 1B).

Populations living in the most contrasted temperature environments showed significant variation in phenotypic traits. For example, individuals from the Valencia population (Spain) fledged on average 3.5 less offspring than those from the Zvenigorod population (Russia) (Supporting Information 1). The mean laying date in the Oulu population (Finland) occurred 1 month later than in the Mediterranean populations. Regarding body mass, the only morphological trait which is not influenced by differences in measurement methods (see *Methods* for details), we found that Spanish individuals were more than 1.7 g lighter than their Russian counterparts. All statistical tests on mean differences are provided in Supporting Information 1 (Supplementary Tables S2–S7).

### Variation in life history G-matrix linked with climatic conditions

**Figure 2.**
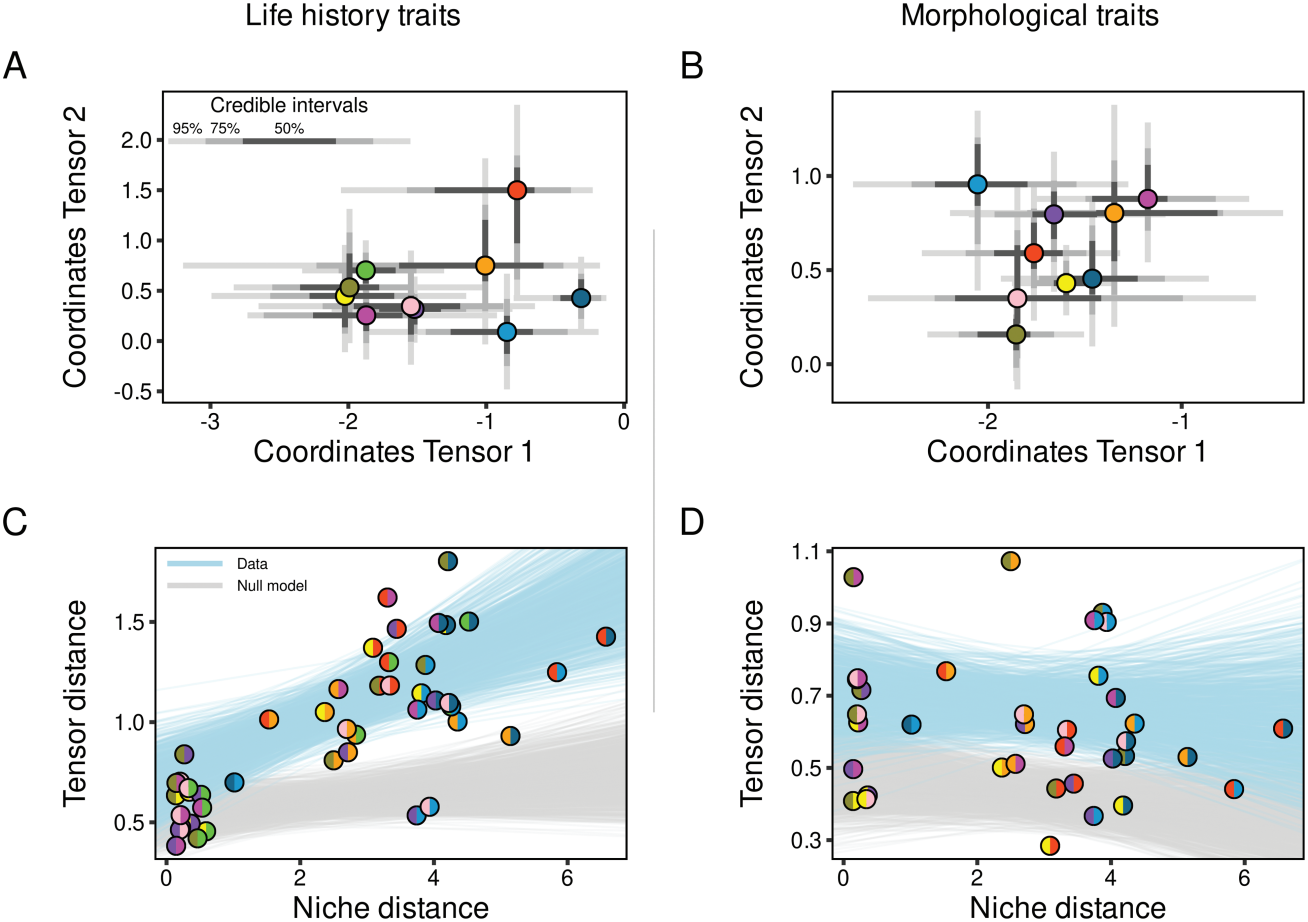
Genetic covariance tensor results. Points in the upper panels represent the posterior modes of the coordinates of each matrix along the two first tensors for (A) life history G-matrices and (B) morphological G-matrices. Colors correspond to the different populations (see Figure 1 for details) and gray bars to the 95%, 75%, and 50% credible intervals (CI). Relation between the Euclidean distance among populations in the two first tensor spaces and the distance of population within their climatic niche are represented for (C) life history and (D) morphological G-matrices. Points represent the distances between pairs of populations (posterior mode for the tensor distances). Colored hemispheres correspond to the population considered for pairwise comparison. The light blue and gray envelops represent the linear regressions realized on posterior distributions of distance estimated using data and null model, respectively. Only regressions with a slope within 95% CI are shown.

**Figure 3.**
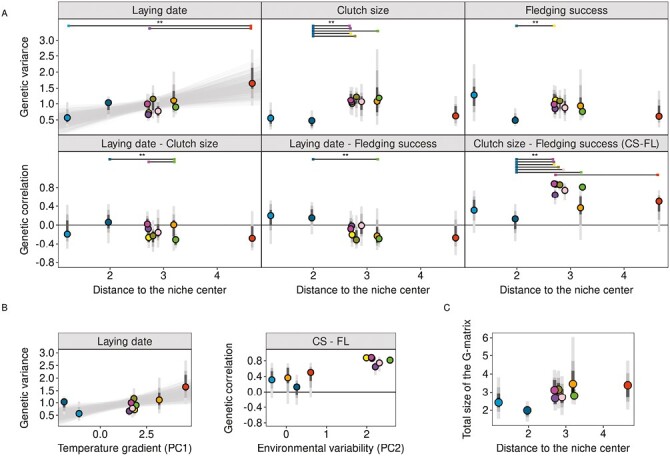
(A) Standardized additive genetic variances and genetic correlations estimated for life history traits. (B) Genetic variance of the laying date and genetic correlation between clutch size and fledging success related with the principal components of the environmental niche (see Supplementary Information 4 for all traits). (C) Volume of the life history G-matrix organized along the distance to the niche center. Points represent the posterior mode estimates filled with colors corresponding to their respective populations and gray bars the 95%, 75%, and 50% credible intervals (CI) (see Figures 1 and 2 for details). On the laying date panel, the gray envelope represents the 95% CI of the significant linear relationship between genetic variance and distance to niche center. Significant differences at a *p*-value of .05 (**) are displayed on top of figures for panel (A).

Given our standardization strategy, results highlighted the relative change in G-matrix across the 10 populations studied. For life history traits, the distance among standardized G-matrices in tensors space can be summarized by the two first eigentensors accounting altogether for 64% of variances among G-matrices (different from the null model at *p*-value of .03 and .09, respectively, Supporting Information 5, Supplementary Figure S5). The distances among G-matrices in tensors space is positively linked with the distances among populations within the climatic niche (Figure 2C, slope_data_[95% CI] = 0.13[0.06:0.21]; Mantel test: *p-*value= .001, *r* = 0.69). This differentiation among G-matrices according to climatic differences was not detected under the null model (slope_null_[95% CI] = 0.02[−0.02:0.09]; Mantel test: *p-*value = .38, *r* = 0.05, slope_data_ is statistically higher than slope_null_ at a *p-*value of .016). This result was robust to the removal of the two most extreme populations (Supporting Information 5, Supplementary Figures S8–S10). The first tensor accounted for 48% [26%:71%] of the variation among G-matrices and revealed that the G-matrices of populations from Southern (Spain, south of France) and Northern Europe (Finland, Russia) tended to be different from the other populations. Along this tensor, the major difference in G-matrices were led by a coordinated change in genetic variance for clutch size and fledging success (the first eigenvector of the first tensor explained 97% of the variation with loadings of −0.74 and −0.64 for clutch size and fledging success, respectively). Significant differences in coordinates on the first tensor were found between the Oulu population (Finland) and the populations located around 50°N (Belgium, Netherlands, England) (Figure 2A, Supporting Information 5, Supplementary Figure S7) reflecting the significantly lower additive genetic variance in clutch size and lower genetic correlation between clutch size and fledging success in the Finnish population (Figure 3A). Overall, the genetic correlation between clutch size and fledging success was lower for populations experiencing higher environmental variability (PC2 in Figures 1 and 3B).

The second tensor accounted for 16% [5%:41%] of the variation among standardized G-matrices and mostly represented a change in additive genetic variance of laying date (the first eigenvector of the second tensor explained 81% of the variation with loadings of 0.93 for the laying date). This tensor highlighted differences between Valencia (Spain) and Zvenigorod (Russia): the additive genetic variance of laying date was significantly higher in the former (Figures 2A and 3A, Supplementary Figure S7). This difference resulted in a significant relation between the genetic variance of laying date and the distance to the niche center (Figure 3A; slope[95% CI] = 0.29[0.05:0.56]) mostly driven by the temperature gradient (Figure 3B; slope[95% CI] = 0.11[0.02:0.23]).

**Figure 4.**
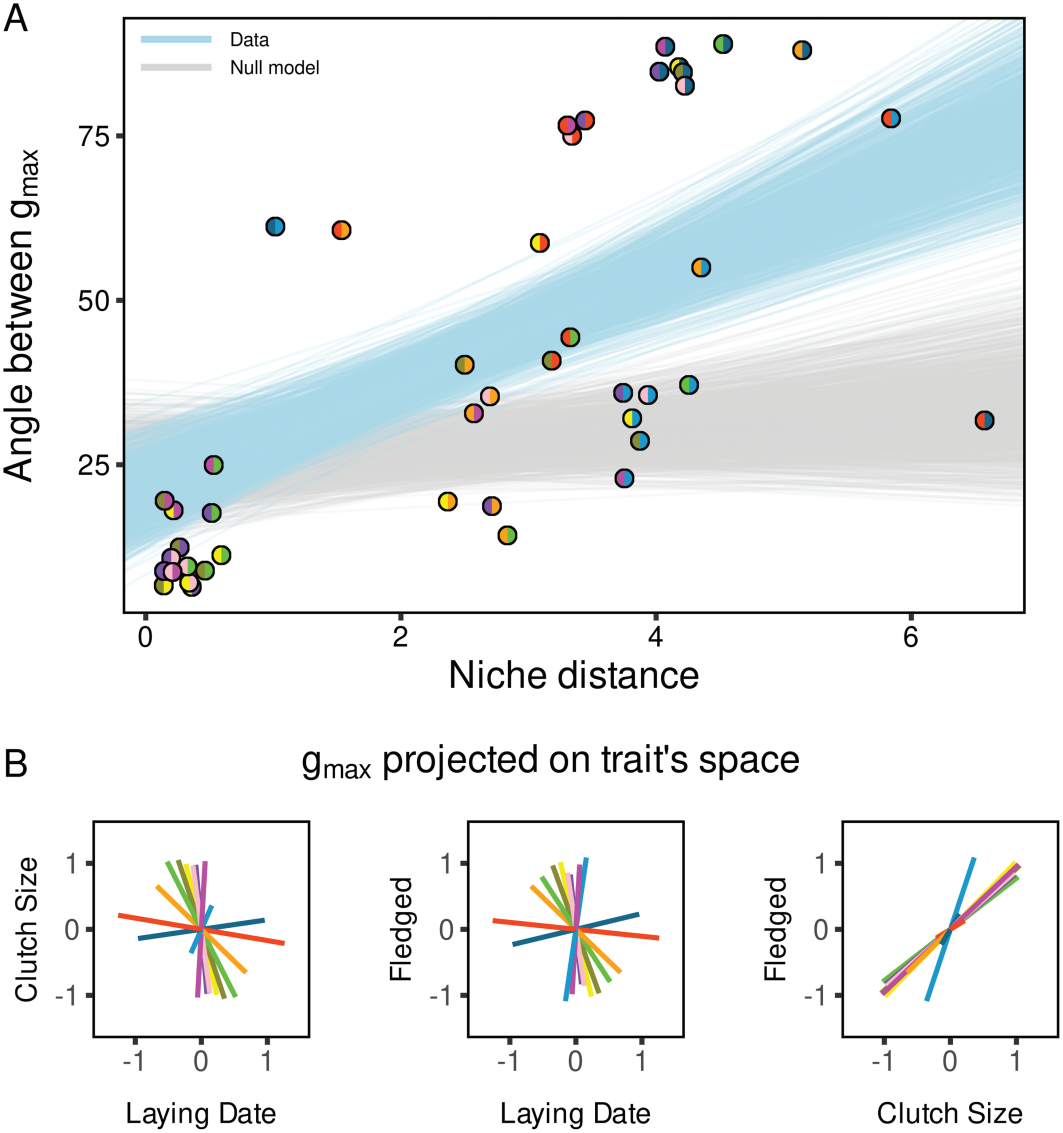
Difference in orientation between G-matrices of life history traits. (A) Relationship between the difference in *g*_max_ direction for each pair of populations and their respective niche distance. Points represent the distances between pairs of populations (posterior mode for the angle distances). Colored hemispheres refer to the population considered for pairwise comparison. The light blue and gray envelops represent the linear regressions realized on posterior distributions of distance estimated using data and null model, respectively. Only regressions with a slope within 95% CI are shown. (B) Projection of the posterior mode of *g*_max_ axes for each population into the additive genetic variance trait space with colors corresponding to their respective populations (see Figure 1 for details).

The angles among populations’ *g*_max_ also increased with the niche distance between populations (Figure 4A) (slope_data_[95% CI] = 8.79[5.40:10.71]; Mantel  **p*-*value = .01, *r* = 0.70; Supporting Information 6). These differences arose because, *g*_max_ was oriented in the direction of laying date for both the Spanish and Finnish populations, of fledging success for the Russian population, and of both clutch size and fledging success in the other populations. As the standardized genetic variances are of the same magnitude for both clutch size and fledging success of these populations, the direction of *g*_max_ is mostly defined by the genetic correlation between these two traits (Figure 4B, bidimensional ellipses are provided in Supporting Information 7). The variances explained by *g*_max_ were similar among all populations (between 0.55 and 0.68, Supporting Information 7, Supplementary Figures S14 and S15). The relation between angles among populations’ *g*_max_ and the niche distance estimated on the null model was not significant (slope_null_[95% CI] = 1.05[−1.52:4.3]; Mantel test: *p-*value = .10, *r* = 0.38, slope_data_ is statistically higher than slope_null_ at a *p-*value of .001).

Although major changes occurred among G-matrices of life history traits, no decrease in their total volume was found from the center to the edge of the niche (slope[95% CI] = 0.31[−0.12:0.28], Figure 3C).

### Variation of morphological G-matrix

For morphological traits, no significant linear relation was found between the distance among standardized G-matrices in the space defined by the two first tensors and the distance among populations within the climatic niche (Figure 2D, slope[95% CI] = −0.008[−0.07:0.04], not significantly different from the null model). Distance among G-matrices in tensors space was based on the two first eigentensors accounting for 61% of variation among G-matrices (different from the null model at *p*-value of .19 and .04, respectively, Supporting Information 5, Supplementary Figure S6).

**Figure 5.**
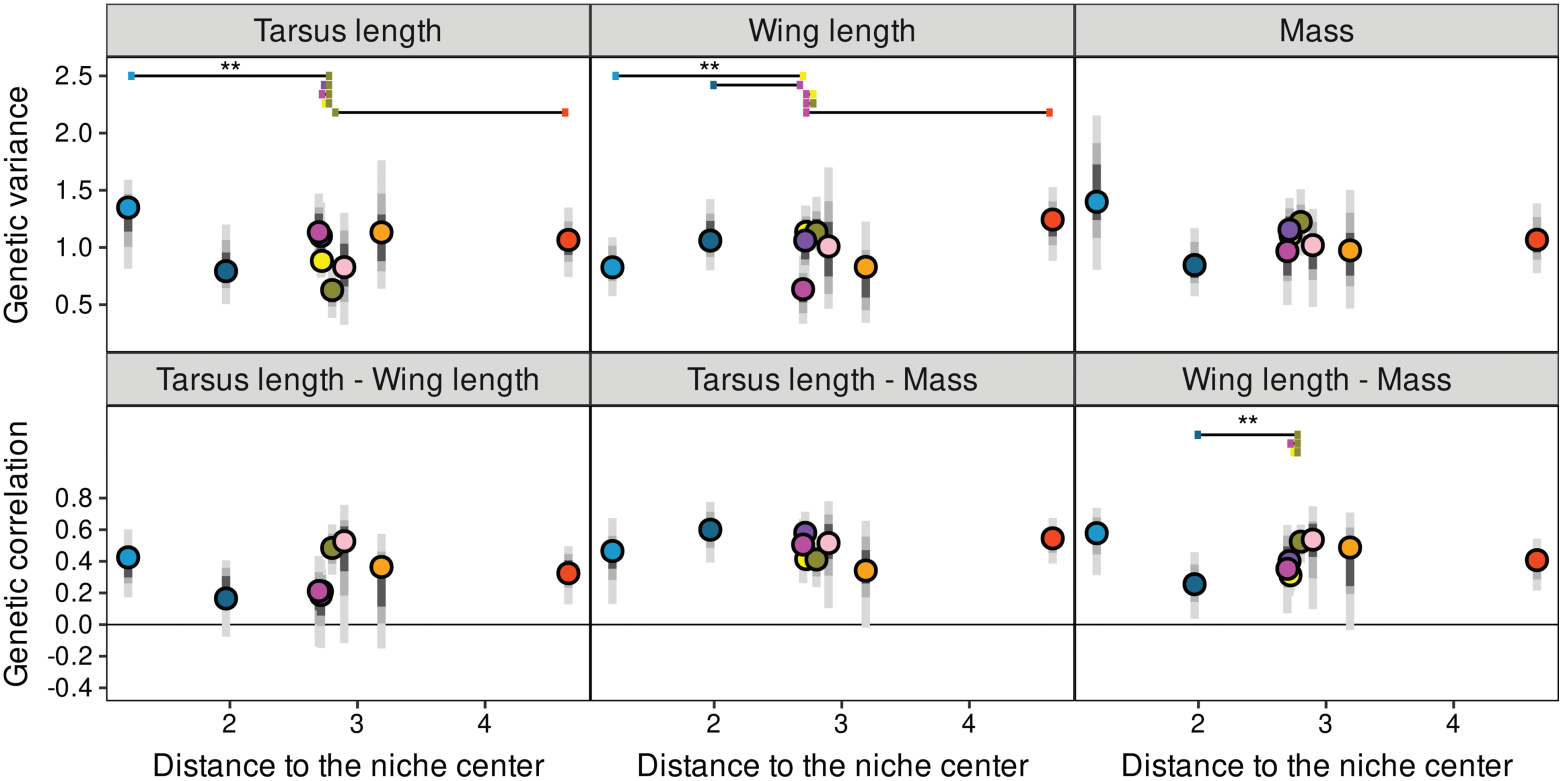
Standardized additive genetic variances and genetic correlations estimated for morphological traits. Details of the legend are provided in Figure 3.

The first tensor accounted for 37% [20%:65%] of the variation among G-matrices. This axis segregated Peerdsbos (Belgium) and Zvenigorod (Russia) from Oosterhout (Netherland) populations (Figure 2B). The differences among the G-matrices of these populations were related to a coordinated change in the genetic variance of wing length, tarsus length, and body mass (the first eigenvector of the first tensor explained 98% of the variation with loadings of both traits, respectively, −0.66, −0.41, and −0.63). The genetic correlation between wing length and body mass was significantly higher in Peerdsbos population than in Oulu, Oosterhout, and Boshoek populations (Figure 5).

The second tensor (24% [11%:47%]) mostly represented a difference in G-matrices between Peerdsbos (Belgium) and several other populations (Valencia, Rouvière, Oosterhout, Zvenigorod, and Hoge Veluwe, Supplementary Figure S7) due to a low additive genetic variance of tarsus length in this population (76% of the variation along the first eigenvector of the second eigentensor; loadings of tarsus length 0.91; Figure 5).

No relation between the angles among populations’ *g*_max_ and the niche distance was found (slope[95% CI] = −0.24[−2.70:1.19], Supplementary Figure S13) as well as between the total size and the distance to the niche center (slope[95% CI] = 0.02[−0.28:0.26], Supplementary Figure S16).

## Discussion

No general consensus can be reached regarding the spatial variation of G-matrices in the wild without evaluating how this variation is environmentally driven. In particular, an important question that needs to be addressed is whether a G-matrix estimated in one population can be extrapolated to other populations or environments (current or future). Using long-term phenotypic data associated with pedigrees in 10 wild populations of great tits, we explored intraspecific variation in G-matrices across the species range. We provide compelling evidence for differences in G-matrices among populations of great tits across Europe for both life history and morphological traits. Interestingly, variation in G-matrices for life history traits (but not morphological traits) could be related to the population’s position along a climatic niche gradient. The major divergence in genetic architecture for life history traits was measured between the populations located close to the 50°N latitude (Belgium, Netherlands, and England) and the other populations located in the Mediterranean and Subarctic climates.

### Variation in the life history G-matrix linked with the bioclimatic niche

#### G-matrix variation among populations

We detected a significant link between the distance between two populations within the species’ climatic niche and their difference in G-matrices for life history traits.

Among all potential mechanisms that may have shaped this relative variation in G-matrices among populations in relation with climate, a response to a change in selection regimes due to environmental variation is a possible explanation. Specifically, we showed that the variation in the G-matrix observed among these 10 wild populations was correlated with the difference in environment for the life history traits, traits most likely to be affected by the change in selection regime due to environmental variation (e.g., Lundblad & Conway, 2020). The large impact of a change in selection regime on the shape of the G-matrix was previously shown in controlled experiments performed on a wide range species (e.g., Cano et al., 2004; Wood & Brodie, 2015). Both the strength and direction of selection vary in space among wild populations of plants and animals especially among contrasted environmental conditions (Siepielski et al., 2017): beyond the question of how G-matrices vary spatially, the open challenge is to identify the nature and strength of environmental variations that alter G-matrix structure such as volume and orientation. In this regard, nonsignificant variation in G-matrices between contrasted environments found in some empirical studies (e.g., reviewed in Arnold et al., 2008) might reveal a lack of contrast between environmental conditions. For example, the lack of variation among life history and morphological G-matrices estimated in island and mainland Mediterranean populations of blue tits (Delahaie et al., 2017) may simply reflect the limited climatic differences that exist between these four Mediterranean populations. On the contrary, significant variation in G-matrices was found between a Mediterranean (Spain) and a Continental (Denmark) populations of barn swallows (*Hirundo rustica*) (Teplitsky et al., 2011). Altogether and despite a lack of generality, these studies and our results tend to show that contrasted climatic conditions, or more generally broad environmental condition, represent a significant factor that partly shape the G-matrix of life history traits. Hence, while G-matrices are not stable, using environment for time substitution could allow to predict more accurate responses to selection, for example, in future warmer climate.

While the spatial variation of G-matrices can partly result from differential selection due to particular local climatic conditions, several alternative scenarios may also explain our results. First, genotype–environment interactions (**G** × **E**) can produce correlation patterns between climatic conditions and G-matrix variation. Recent studies that compared DNA markers among populations of great tits distributed across Europe highlighted low overall genetic differentiation among European populations of great tits except for populations in small Mediterranean islands (i.e., Corsica, Sardinia, and Crete) (Laine et al., 2016; Lemoine et al., 2016; Spurgin et al., 2019). In this context, it is likely that a **G** × **E** interaction may contribute to the variation of G-matrices and produce a pattern that may be similar to a change in allelic frequencies between populations. Disentangling both processes requires common garden experiments, a difficult task for many species, but future studies based on gene expression may help solve this issue. Whether G-matrices differences result from **G** × **E** and/or selection processes, this study highlights the importance of climatic conditions on the structure of the G-matrix and consequences for predicting responses to selection in these environments.

Second, genetic drift and founder effects may lead to large G-matrix variation among wild populations (e.g., Paccard et al., 2016). The low overall genetic differentiation between populations at the European scale and generally large effective population sizes suggest that drift may not be a determining factor for most of the differences observed between our G-matrix (Kvist et al., 1999; Laine et al., 2016). Nevertheless, significant genetic differentiation has been reported between Spanish populations and other populations, suggesting that this population may be more isolated and experienced higher selective pressure or stronger drift (Lemoine et al., 2016). Third, indirect genetic effects (i.e., parental care, social behavior) may alter the distribution of phenotypic variance within and among families and affect the estimation of additive genetic variance when using animal models (Kruuk & Hadfield, 2007). Consequently, our results might also partly reflect a potential variation across Europe of some indirect genetic effects in great tits such as parental effect (Garant et al., 2008; Postma, 2005). Unfortunately, a precise partitioning of the additive genetic and indirect genetic effects was not possible for all the populations due to lack of sufficient data.

#### G-matrix variation along niche gradient

Contrary to expectations, no coordinated decrease in genetic variance was found toward the edge of the environmental niche, suggesting a limited impact of genetic drift in the edge populations for this species. Edge effects on evolutionary potential have been previously found in European wild birds when comparing trait evolvabilities along climatic gradients for 12 species (Martínez-Padilla et al., 2017). Our result is however in line with the overall low genetic differentiation among European populations of great tits populations in this study suggesting low-drift effect and that none of the populations here is truly at the edge of the climatic niche. Actually, we even found a significant increase in the additive genetic variance of laying date along a temperature gradient, with the highest genetic variance in the Spanish population, closest to the edge of the great tits niche. This pattern of increase in genetic variance from the center to the niche margin has been predicted to arise when gene flow between populations living in contrasted environments with different phenotypic optima (Polechová, 2018; Polechová & Barton, 2015). It could be the case here as laying date is generally considered to display an optimum in great tits (Gamelon et al., 2018; De Villemereuil et al., 2020). Under this theoretical model, genetic variance is expected to decrease at the extreme margin of the niche only, where the effects of drift and demographic stochasticity become predominant.

We did not detect a consistent change of all (co)-variance components along the niche gradient, and this should not be expected unless selection vary in a coordinated way across the climatic range for all traits. Moreover, the interpretation of variations of each element of the G-matrix may be misleading when the genetic correlation between traits varies greatly from one population to another (Blows, 2007). Here, we found strong genetic correlations between clutch size and fledging success only in populations located around 50°N latitude, likely defined by an environmental variability gradient. Thus, a finer understanding of change in genetic variance and covariance of the clutch size and fledging success will require to compare the phenotypic optima and selection strength, including correlational selection, across populations.

### Variation in the G-matrix for morphological traits

While the G-matrix for morphological traits differed among populations, no link with a climatic gradient was found, as opposed to our findings for the G-matrix for life history traits. According to the tensor analysis, the standardized morphological G-matrix was most different between the Russian populations and one of the Belgian and the Dutch populations (Figure 2B) due to both a change in genetic variances and genetic correlations between traits. These results suggested that factors other than climatic conditions were responsible for this variation in morphological genetic (co)variances. Overall, the stability of the genetic architecture was higher for morphological traits than life history traits (Supplementary Figures S14 and S15).

Several nonmutually exclusive explanations may be involved in the relative stability of the morphological G-matrix compared to the life history G-matrix. First, morphological traits have often been shown to be under stabilizing selection in birds (e.g., mass: Covas et al., 2002; tarsus length: Alatalo & Lundberg, 1986) and the constraint imposed by flight traits is high and possibly shared across populations independently of weather conditions. The relative stability of the G-matrix of morphological traits in our study was in line with predictions based on the expected stability of selection pressures on these traits (Arnold et al., 2001). Second, the existence of unmeasured traits strongly correlated with focal morphological traits can constrain and even prevent structural changes in G-matrix. Indeed, strong pleiotropic genes or selective correlation between traits could constrain and stabilize the G-matrix (Chantepie & Chevin, 2020; Jones et al., 2003). A further understanding of variation in the G-matrix of morphological traits would require the comparison of populations experimenting substantially different selection regimes on morphology. Ecological specialization has, for example, been shown to shape the G-matrix for morphological traits across seven *Anolis* lizards species (McGlothlin et al., 2018). A recent study showed that great tits that live in cities are smaller than forest birds (Caizergues et al., 2021), which could be due to a change in the selection regime and lead to a variation of the G-matrix. Selection due to migration might also act on morphological traits of passerines (Vágási et al., 2016). Although Northern populations have been shown to be partial residents migrating irregularly (Kvist et al., 1999), the lack of longitudinal data with pedigree and migration information for these populations preclude actual testing of the impact of migration on the G-matrix of morphological traits. However, undertaking comparisons of G-matrices in these ecological specialization contexts would help to test our hypothesis that G-matrix of morphological traits is only relatively insensitive to variation in climatic conditions but not to all environmental variations.

## Conclusion

The increasing number of long-term datasets collected including both phenotypic and pedigree information in wild populations opens new perspectives to assess the link between evolutionary processes and environmental conditions. This study shows that assuming a constant G-matrix among populations may not be a valid approximation for life history traits and to a lesser extent for morphological traits. Furthermore, the heterogeneity of climatic conditions across the spatial distribution of great tits partially explained the differences in G-matrices among populations for life history traits which open new perspectives in understanding and predicting the adaptive response of life history traits to global changes at the species level.

## Supplementary Material

qrad067_suppl_Supplementary_Material

## Data Availability

Data are available upon request at https://spibirds.org. This hub acts as a central contact point for accessing the data, ensures quality checks on the data to correct for any mistakes in the datasets and warns users about potential issues when using the data. This hub is maintained by the Netherlands Institute of Ecology. Full output of animal models, unstandardized G-matrix, standardized G-matrix, unstandardized G-matrix estimated with the null models, and standardized G-matrix estimated with the null models are available at https://doi.org/10.6084/m9.figshare.24783900.v1. Code for null model simulation is available at https://github.com/schantepie/NullGmatrix/.
