# Supplementary material for: Divergence in evolutionary potential of life history traits among wild populations is predicted by differences in climatic conditions
Source: Evol Lett. 2024 Feb 1;8(1):29–42. doi: 10.1093/evlett/qrad067 (PMC10872211; doi:10.1093/evlett/qrad067)
Supplement: qrad067_suppl_Supplementary_Material [file qrad067_suppl_supplementary_material.pdf]

Online appendix to: Divergence in evolutionary potential of life-history traits among wild populations is predicted by differences in climatic conditions

Stéphane Chantepie, Anne Charmantier, Boris Delahaie, Frank Adriaensen, Erik Matthysen, Marcel E. Visser, Elena Álvarez, Emilio Barba, Markku Orell, Ben Sheldon, Elena Ivankina, Anvar Kerimov, Sébastien Lavergne, Céline Teplitsky

Supporting Information 1 : Information about populations and phenotypic traits. Statistical tests comparing the variation of phenotypic mean between populations for all traits

Table S1: Populations characteristics 3
Table S2-S7: Test of mean differences among populations 4-7

Supporting Information 2 : Pedigree information and parameters fitted for each animal model

Tables S8-S9: Sample size and pedigree information for life-history traits and morphological traits 8
Table S10: Fixed and random effects used in the animal models for each trait in each population 9-10

Supporting Information 3 : Complementary information about standardisation

Supporting Information 4 : Values of standardised G-matrix, unstandardised G-matrix, P-matrix and heritability

Tables S11-S12: Unstandardised G-matrix for life-history and morphological traits 12
Tables S13-S14: Standardised G-matrix for life-history and morphological traits 13
Tables S15-S16: P-matrices for life-history and morphological traits 14
Table S17: Estimates of heritability 15
Figures S1-S2: Statistical differences between (a) additive genetic values and (b) genetic correlations for life-history and morphological traits 15-16
Figure S3: Standardized additive genetic variances and genetic correlations according to PC1 of the climatic niche 17
Figure S4: Standardized additive genetic variances and genetic correlations according to PC2 of the climatic niche 18

Supporting Information 5 : Details on tensor method and results

Figure S5-S6: Variance accounted for by each eigentensor with comparison of the observed G-matrices and the null G-matrices for life-history and morphological traits . . . 20-21
Table S18-S19: P-value of the differences between the eigentensors estimated from the observed data and the eigentensors estimated from null model for life-history traits . . . 20-21
Tables S20-S21: Coordinates of populations with 95% Credible Interval (CI) on the first and second tensors for G-matrices of life-history and mortphologicla traits . . . 22
Figure S7: Statistical differences in coordinate values among populations for life-history and morphological traits . . . 23
Tables S22-S23: Results of the tensor analysis on standardised G-matrices for life-history and morphological traits . . . 24
Figures S8-S10: Results of the tensor analysis when removing the extreme populations . . . 25-27

Supporting Information 6: Angle analysis details and results

Tables S24-S25: Angle values between g_{max} for each pair of populations for the G-matrix of life-history and morphological traits . . . 29-31
Figure S11-S12: Statistical differences between g_{max} angles for the G-matrix of life-history and morphological traits . . . 30-32
Figures S13: Difference in orientation between G-matrices of morphological traits 33

Supporting Information 7: Representations of the G matrices in the form of bidimensional ellipse , volume and eccentricity estimates

Figure S14-15: Standardized G-matrix in the form of bidimensional ellipse for morphological traits . . . 34-35
Figure S16: Total size of the G-matrices of life-history traits according to the distance of population to the niche center . . . 36
Table S26: Percent of variance explained by g_{max} for life-history and morphological G-matrix (eccentricity) . . . 37

References

Supporting Information 1: Information about populations and phenotypic traits. Statistical tests comparing the variation of phenotypic mean between populations for all traits

1.1 Populations locations and summary statistics

Table S1: Geographical coordinates of the ten great tit populations along with their mean trait values and standard deviations for the life-history and morphological traits. Body mass is in grams, tarsus and wing lengths are in millimeters. U.K. is used for United Kingdom. [a] measured with slightly different methods (see materials and methods).

Population	Country	Latitude	Longitude	Sample period	Mean trait value (standard deviation)					
					Clutch size	Laying date	Fledging success	Body mass	Tarsus length	Wing length
Oulu	Finland	65°03'N	25°26'E	1975-2015	9.48 (1.56)	135.66 (5.82)	6.50 (2.35)	18.15 (1.07)	19.77 (0.73)	76.19 (2.50)
Zvenigorod	Russia	55°44'N	36°51'E	1992-2016	10.86 (1.42)	117.75 (5.55)	9.15 (2.41)	18.72 (1.29)	19.38 (0.68)	75.20 (2.26)
Hoge Veluwe	Netherlands	52°05'N	05°50'E	1955-2014	9.35 (1.83)	112.01 (7.68)	6.81 (2.60)	18.10 (1.11)	19.82 (0.65)	56.37[a] (2.05)
Oosterhout	Netherlands	51°55'N	05°50'E	1957-2014	8.94 (1.56)	108.16 (7.91)	8.15 (1.90)	18.05 (1.08)	19.73 (0.69)	56.40[a] (2.23)
Liesbos	Netherland	51°35'N	04°40'E	1955-2014	9.85 (1.84)	111.05 (7.63)	8.58 (2.21)	18.21 (1.18)	19.92 (0.84)	56.23[a] (2.42)
Peerdsbos	Belgium	51°16'N	04°29'E	1979-2014	8.96 (1.64)	110.38 (8.80)	7.95 (2.06)	17.57 (0.86)	17.46[a] (0.67)	74.12 (2.23)
Boshoek	Belgium	51°08'N	04°32'E	1994-2014	9.05 (1.58)	100.89 (8.08)	7.93 (2.01)	17.44 (0.81)	17.54[a] (0.72)	74.38 (2.40)
Wytham Woods	U.K.	51°46'N	01°20'W	1961-2004	8.72 (1.64)	116.29 (8.85)	7.52 (2.20)	**	**	**
Rouvière	France	43°40'N	03°40'E	1991-2015	9.07 (1.99)	98.80 (8.38)	7.18 (2.45)	17.52 (1.04)	19.66 (0.61)	74.96 (2.49)
Valencia	Spain	39°42'N	0°15'W	1993-2013	8.09 (1.37)	102.65 (7.41)	5.65 (1.83)	17.02 (0.96)	19.57 (0.67)	72.03 (2.40)

1.2 Phenotypic comparisons across sites

The results presented below include for each trait a linear mixed model to assess whether the trait varies across populations. Population names were used as fixed effects all trait and sex were fitted for morphological traits (mass, wing length, tarsus length). The year of the measurement and the identity of the individual were fitted as random effects for all traits. We used the function 'lmer' from the R-package 'lmerTest' to perform the analyses. Degree of freedom and significance of fixed effects were based on the Satterthwaite's method (Kuznetsova et al. 2017).

All traits vary significantly across populations (Table S2-S7).

Table S2: Results of the linear mixed model realized on the clutch size trait. t value corresponds to the t-test value fitted using Satterthwaite's method. Significativity codes for p-values (Pr(>|t|)): 0 '***', <0.001 '**', <0.01 '*', <0.05 '.'. Number of observations: 25176. Number of individuals (id): 16296. Number of years: 62.

**Random effect**	**Variance**	**Standard deviation**			
id	1.1808	1.0867			
year	0.2836	0.5326			
Residual	1.4363	1.1984			
**Fixed effect**	**Estimate**	**Standard error**	**Degree of freedom**	**t value**	**Pr(>$\lvert$t$\rvert$)**
(Intercept)	9.319	0.08057	105.4	115.66	<2E-16***
Hoge Veluwe	0.1021	0.04949	14190	2.063	0.0391*
Liesbos	0.4191	0.06227	15610	6.73	1.75E-11***
Oosterhout	-0.2743	0.06175	13940	-4.443	8.96E-06***
Oulu	0.3621	0.05722	15000	6.328	2.56E-10***
Peerdbos	-0.3383	0.06877	14120	-4.919	8.81E-07***
Rouvière	0.08601	0.08761	17970	0.982	0.3263
Valencia	-0.9417	0.06661	14310	-14.137	<2E-16***
Wytham Woods	-0.6145	0.04879	14210	-12.593	<2E-16***
Zvenigorod	1.782	0.09057	17990	19.679	<2E-16***

Table S3: Results of the linear mixed model realized on the number of fledged trait. t value corresponds to the t-test value fitted using Satterthwaite's method. Significativity codes for p-values (Pr(>|t|)): 0 '***', <0.001 '**', <0.01 '*', <0.05 '.'. Number of observations: 21545. Number of individuals (id): 14350. Number of years: 62.

**Random effect**	**Variance**	**Standard deviation**			
id	1.0248	1.0123			
year	0.4716	0.6867			
Residual	3.7276	1.9307			
**Fixed effect**	**Estimate**	**Standard error**	**Degree of freedom**	**t value**	**Pr(>|t|)**
(Intercept)	7.951	0.1038	105	76.592	<2E-16***
Hoge Veluwe	-1.155	0.06488	10920	-17.795	<2E-16***
Liesbos	0.5176	0.08177	12830	6.33	0.0131*
Oosterhout	0.1981	0.07984	10390	2.481	<2E-16***
Oulu	-1.448	0.07614	12110	-19.019	<2E-16***
Peerdbos	-0.1377	0.08786	10520	-1.567	0.1171
Rouvière	-0.6034	0.1341	15340	-4.501	6.82E-06***
Valencia	-2.627	0.1385	11230	-18.97	<2E-16***
Wytham Woods	-0.5139	0.06327	10760	-8.122	5.09E-16***
Zvenigorod	1.363	0.1168	16000	11.666	<2E-16***

Table S4: Results of the linear mixed model realized on the laying date trait. t value corresponds to the t-test value fitted using Satterthwaite's method. Significativity codes for p-values (Pr(>|t|)): 0 '***', <0.001 '**', <0.01 '*', <0.05 '.'. Number of observations: 25252. Number of individuals (id): 16346. Number of years: 62.

**Random effect**	**Variance**	**Standard deviation**			
id	10.51	3.242			
year	27.36	5.231			
Residual	31.99	5.656			
**Fixed effect**	**Estimate**	**Standard error**	**Degree of freedom**	**t value**	**Pr(>|t|)**
(Intercept)	104.4902	0.6851	67.7479	152.526	<2E-16***
Hoge Veluwe	7.9004	0.1888	12943.3485	41.844	<2E-16***
Liesbos	6.4465	0.2413	15385.5559	26.715	<2E-16***
Oosterhout	4.8583	0.2347	12494.7172	20.696	<2E-16***
Oulu	32.748	0.2192	14060.6044	149.429	<2E-16***
Peerdbos	6.869	0.262	12826.4167	26.222	<2E-16***
Rouvière	-2.0195	0.3453	19025.5233	-5.849	5.03E-09***
Valencia	1.0963	0.2525	13033.8869	4.341	1.43E-05***
Wytham Woods	11.9625	0.1862	12942.646	64.239	<2E-16***
Zvenigorod	15.7362	0.3474	19038.5743	45.293	<2E-16***

Table S5: Results of the linear mixed model realized on the mass trait. t value corresponds to the t-test value fitted using Satterthwaite's method. Significativity codes for p-values (Pr(>|t|)): 0 '***', <0.001 '**', <0.01 '*', <0.05 '.'. Number of observations: 56187. Number of individuals (id): 20764. Number of years: 61.

**Random effect**	**Variance**	**Standard deviation**			
id	0.47816	0.6915			
year	0.03358	0.1833			
Residual	0.47632	0.6902			
**Fixed effect**	**Estimate**	**Standard error**	**Degree of freedom**	**t value**	**Pr(>|t|)**
(Intercept)	17.16	0.03142	128.5	546.217	<2E-16***
Hoge Veluwe	0.4857	0.02164	20180	22.445	<2E-16***
Liesbos	0.6486	0.03035	22810	21.368	<2E-16***
Oosterhout	0.5014	0.02758	19670	18.177	<2E-16***
Oulu	0.6687	0.02536	25700	26.372	<2E-16***
Peerdbos	0.07707	0.02979	23570	2.587	0.00969**
Rouvière	-0.01116	0.04358	23500	-0.256	0.79792
Valencia	-0.4162	0.02743	23360	-15.172	<2E-16***
Zvenigorod	1.207	0.02867	23980	42.121	<2E-16***
Sex (Male)	0.6867	0.01213	19990	56.621	<2E-16***

Table S6: Results of the linear mixed model realized on the wing length trait. t value corresponds to the t-test value fitted using Satterthwaite's method. Significativity codes for p-values (Pr(>|t|)): 0 '***', <0.001 '**', <0.01 '*', <0.05 '.'. Number of observations: 25323. Number of individuals (id): 16033. Number of years: 41.

**Random effect**	**Variance**	**Standard deviation**			
id	1.48744	1.2196			
year	0.09075	0.3012			
Residual	1.76054	1.3269			
**Fixed effect**	**Estimate**	**Standard error**	**Degree of freedom**	**t value**	**Pr(>|t|)**
(Intercept)	72.9	0.06105	80.04	1193.942	<2E-16***
Hoge Veluwe	-17.96	0.04415	14140	-406.841	<2E-16***
Liesbos	-18.35	0.07507	15960	-244.421	<2E-16***
Oosterhout	-17.75	0.06101	14200	-291.04	<2E-16***
Oulu	1.857	0.04859	12580	38.218	<2E-16***
Peerdbos	-0.3212	0.05737	10790	-5.599	2.21E-8***
Rouvière	0.5711	0.0803	14330	7.111	1.2E-12***
Valencia	-2.156	0.05029	14520	-42.869	<2E-16***
Zvenigorod	0.5511	0.05089	16000	10.828	<2E-16***
Sex (Male)	2.83	0.02646	15370	106.952	<2E-16***

Table S7: Results of the linear mixed model realized on tarsus length trait. t value corresponds to the t-test value fitted using Satterthwaite's method. Significativity codes for p-values (Pr(>|t|)): 0 '***', <0.001 '**', <0.01 '*', <0.05 '.'. Number of observations: 31655. Number of individuals (id): 18554. Number of years: 42.

**Random effect**	**Variance**	**Standard deviation**			
id	0.2748	0.5242			
year	0.01591	0.1262			
Residual	0.12135	0.3484			
**Fixed effect**	**Estimate**	**Standard error**	**Degree of freedom**	**t value**	**Pr(>|t|)**
(Intercept)	17.31	0.02365	74.66	731.854	<2E-16***
Hoge Veluwe	2.22	0.01519	17650	146.175	<2E-16***
Liesbos	2.356	0.02181	19020	108.044	<2E-16***
Oosterhout	2.167	0.01999	17720	108.409	<2E-16***
Oulu	2.172	0.01702	18810	127.6	<2E-16***
Peerdbos	-0.1179	0.02009	18050	-5.869	4.47E-9***
Rouvière	2.052	0.02928	17990	70.089	<2E-16***
Valencia	2.012	0.01834	17920	109.733	<2E-16***
Zvenigorod	1.753	0.01892	18980	92.673	<2E-16***
Sex (Male)	0.5582	0.008883	18000	62.836	<2E-16***

Supporting Information 2: Pedigree information and parameters fitted for each animal model

For each population, the standard pedigree statistics were calculated for data set only containing informative individuals, resulting in one pedigree for life-history traits (Table S8) and another for morphological traits (Table S9). Because the effects fitted in each model depended on the populations and traits, we also give a summary of the fixed and random effects included in the animal models (Table S10).

Table S8: Sample size and pedigree information for life-history traits.

	Oulu	Zvenigorod	Hoge Veluwe	Liesbos	Oosterhout	Boshoek	Peerdbos	Wytham Woods	Rouvière	Valencia
individuals	1596	446	4258	1430	1295	1755	872	6847	469	948
maternal links	263	17	1514	312	644	975	339	3258	46	306
paternal links	117	45	1067	179	476	751	166	3863	46	199
full sibs	36	2	450	68	239	413	53	1849	10	93
maternal sibs	54	5	744	112	524	594	97	2648	12	141
maternal half sibs	18	3	294	44	285	181	44	799	2	48
paternal sibs	48	10	693	109	367	583	89	2822	11	112
paternal half sibs	12	8	243	41	128	170	36	973	1	19
maternal grandmothers	50	3	477	79	330	421	81	1829	4	92
maternal grandfathers	25	14	351	47	245	321	41	2074	4	49
paternal grandmothers	41	2	676	98	334	437	96	1773	8	90
paternal grandfathers	19	7	529	57	245	340	42	2076	8	66
maximum pedigree depth	8	4	21	12	20	12	10	33	4	8

Table S9: Sample size and pedigree information for morphological traits.

	Oulu	Zvenigorod	Hoge Veluwe	Liesbos	Oosterhout	Boshoek	Peerdbos	Wytham Woods	Rouvière	Valencia
individuals	3013	2100	7447	2273	2063	2754	1622	x	560	1882
maternal links	499	148	1531	320	644	1520	665	x	95	598
paternal links	491	145	1079	180	476	1444	607	x	86	566
full sibs	130	42	457	69	239	837	181	x	31	277
maternal sibs	198	60	753	115	524	1275	366	x	41	392
maternal half sibs	68	18	296	46	285	438	185	x	10	115
paternal sibs	186	68	706	112	367	1239	279	x	41	377
paternal half sibs	56	26	249	43	128	402	98	x	10	100
maternal grandmothers	99	29	481	81	330	655	161	x	12	183
maternal grandfathers	96	32	353	47	245	638	150	x	12	176
paternal grandmothers	97	19	685	99	334	667	191	x	17	188
paternal grandfathers	95	20	534	58	245	639	178	x	16	188
maximum pedigree depth	8	5	21	12	20	12	10	x	5	8

Table S10: Fixed and random effects used in the animal models for each trait in each population. Values can be found in the supporting data linked to the study.

**Oulu**		**Clutch size**	**Laying date**	**Number of fledged**	**Tarsus length**	**Wing length**	**Body mass**
**Fixed effect**							
	age (2 classes*)	x	x	x	x	x	x
	sex				x	x	x
	age x sex				x	x	x
	ordinal date (quadratic)					x	x
**Random effect**							
	additive genetic variance	x	x	x	x	x	x
	permanent environment	x	x	x	x	x	x
	year	x	x	x	x	x	x
	observer ID				x	x	

**Zvenigorod**		**Clutch size**	**Laying date**	**Number of fledged**	**Tarsus length**	**Wing length**	**Body mass**
**Fixed effect**							
	age (2 classes*)	x	x	x	x	x	x
	sex				x	x	x
	age x sex				x	x	x
	date (4 classes**)				x	x	x
**Random effect**							
	additive genetic variance	x	x	x	x	x	x
	permanent environment	x	x	x	x	x	x
	year	x	x	x	x	x	x

**Hoge Veluwe**		**Clutch size**	**Laying date**	**Number of fledged**	**Tarsus length**	**Wing length**	**Body mass**
**Fixed effect**							
	age (quadratic)	x	x	x	x	x	x
	sex				x	x	x
	ordinal date (quadratic)					x	x
**Random effect**							
	additive genetic variance	x	x	x	x	x	x
	permanent environment	x	x	x	x	x	x
	year	x	x	x	x	x	x

**Liesbos**		**Clutch size**	**Laying date**	**Number of fledged**	**Tarsus length**	**Wing length**	**Body mass**
**Fixed effect**							
	age (quadratic)	x	x	x	x	x	x
	sex				x	x	x
	ordinal date (cubic)					x	x
**Random effect**							
	additive genetic variance	x	x	x	x	x	x
	permanent environment	x	x	x	x	x	x
	year	x	x	x	x	x	x

**Oosterhout**		**Clutch size**	**Laying date**	**Number of fledged**	**Tarsus length**	**Wing length**	**Body mass**
**Fixed effect**							
	age (quadratic)	x	x	x	x	x	x
	sex				x	x	x
	ordinal date (cubic)					x	x
**Random effect**							
	additive genetic variance	x	x	x	x	x	x
	permanent environment	x	x	x	x	x	x
	year	x	x	x	x	x	x

**Boshoek**		**Clutch size**	**Laying date**	**Number of fledged**	**Tarsus length**	**Wing length**	**Body mass**
**Fixed effect**							
	age (quadratic)	x	x	x	x	x	x
	sex				x	x	x
	age (quadratic) x sex				x	x	x
	ordinal date (cubic)					x	x
**Random effect**							
	additive genetic variance	x	x	x	x	x	x
	permanent environment	x	x	x	x	x	x
	year	x	x	x	x	x	x
	observer ID				x	x	

Continued on the next page.

Table S10 (Continued): Fixed and random effects used in the animal models for each trait in each population. Values can be found in the supporting data linked to the study.2 classes of age corresponds first year of breeding and older breeder. 4 classes of date refers to the season of collect. 4 classes of age corresponds to EURING age codes between 4 and 7.

**Peerdbos**		**Clutch size**	**Laying date**	**Number of fledged**	**Tarsus length**	**Wing length**	**Body mass**
**Fixed effect**							
	age (quadratic)	x	x	x	x	x	x
	sex				x	x	x
	age (quadratic) x sex				x	x	x
	ordinal date (cubic)					x	x
**Random effect**							
	additive genetic variance	x	x	x	x	x	x
	permanent environment	x	x	x	x	x	x
	year	x	x	x	x	x	x
	observer ID				x	x	

**Wytham Woods**		**Clutch size**	**Laying date**	**Number of fledged**
**Fixed effect**				
	age (quadratic)	x	x	x
**Random effect**				
	additive genetic variance	x	x	x
	permanent environment	x	x	x
	year	x	x	x

**Rouvière**		**Clutch size**	**Laying date**	**Number of fledged**	**Tarsus length**	**Wing length**	**Body mass**
**Fixed effect**							
	age (quadratic)	x	x	x	x	x	x
	sex				x	x	x
	age (quadratic) x sex				x	x	x
	ordinal date (cubic)					x	x
**Random effect**							
	additive genetic variance	x	x	x	x	x	x
	permanent environment	x	x	x	x	x	x
	year	x	x	x	x	x	x
	observer ID				x	x	

**Valencia**		**Clutch size**	**Laying date**	**Number of fledged**	**Tarsus length**	**Wing length**	**Body mass**
**Fixed effect**							
	age (4 classes***)	x	x	x	x	x	x
	sex				x	x	x
	ordinal date (cubic)					x	x
**Random effect**							
	additive genetic variance	x	x	x	x	x	x
	permanent environment	x	x	x	x	x	x
	year	x	x	x	x	x	x

* The ’2 classes’ of age correspond first year of breeding and older breeder.
** The ’4 classes’ of date refers to the season of collect.
*** The ’4 classes’ of age correspond to EURING age codes between 4 and 7.

Supporting Information 3: Complementary information about standardisation

We standardized the G-matrices by the inter-population mean of the additive genetic variance as :

$$\mathbf{G_{va}} = \mathbf{G} \oslash (\sqrt{Va}\sqrt{Va}')$$

where $\oslash$ denotes element-wise division, $\sqrt{Va}$ is a vector of trait size with the standard deviation of the mean genetic variance for each trait. $'$ is used for transpose. Thus, the ij^{th} element of the G-matrix is standardized by division by division by the product of the means of $\sqrt{Va}$ of traits i and j.

We estimated the inter-population mean of additive genetic variance in two steps. First, for each population and each of the 1000 posterior samples of the G-matrix, we drew 1000 multivariate normal estimates (these estimates correspond to the breeding values of founders). Second, all breeding values were merged and the variance in breeding values estimated. This corresponds to the additive genetic variance if all individuals of the different populations were part of a single population (i.e. the hypothetical species genetic variance). $\sqrt{Va}$ were estimated at 2.56 for laying date, 0.78 the clutch size, 0.81 for number of fledged, 0.37 for tarsus length, 0.92 for wing length and 0.54 for mass.

For a given trait, the genetic variances of all populations are standardized by the same constant. Then, the relative variations among standardized values of Va displayed in Fig. 2 of the manuscript are unchanged when compared to the original scale of Va. Consequently, the interpretation of our results when realized within a trait remains straightforward as this standardisation keeps the pattern similar to the unscaled traits. *For each trait, we can interpret the scaled variables as the proportion of additive genetic variation relative to the species' average.*

Genetic covariances were divided by the square root of the product of mean traits additive genetic variances to preserve the correlation among traits. Standardizing the covariances by the products of the square roots is similar to classical standardization by phenotypic variance G_{vp} as described in Houle et al (2008):

$$\mathbf{G_{vp}} = \mathbf{G} \oslash (\sigma\sigma')$$

σ is a vector of phenotypic standard deviations. As $\sigma = \sqrt{Vp}$, we have :

$$\mathbf{G_{vp}} = \mathbf{G} \oslash (\sqrt{Vp}\sqrt{Vp}')$$

Overall, this standardization is focusing on *preserving the relative variation in variance and covariance of traits among populations in order to adequately describe the variations among G-matrix but was not intended to give insights on the difference in absolute evolvability among traits.*.

Hansen et al. (2008, 2011) and Houle et al. (2012) provided an extensive theoretical framework for the standardisation of genetic values. They showed that the mean trait standardized Va (or evolvability) has good mathematical properties and strong evolutionary meaning. One potential drawback of the mean trait standardisation remains that it can be sensitive to very low values of the mean. In our study, some differences in mean were due to measurement methods and it appeared that these differences in means suspiciously drove all the variation in evolvability among populations (results not shown). Moreover, recalling that the mean trait standardisation cannot be used on laying date, we did not use such standardisation in this study. Nevertheless, we provide in this supporting information the unstandardised G-matrix, the mean of the traits and heritability to allow evolvabilities to be derived and used if needed for future meta-analyses (Supporting Information 4).

Supporting Information 4: Values of standardised G-matrix, unstandardised G-matrix, P-matrix and heritability

Table S11: Unstandardised G-matrix for life-history traits. Va, Cov and Correl respectively correspond to additive genetic variance, genetic covariance and genetic correlation. Values are posterior modes with their 95% credible intervals.

Unstandardized values	Oulu	Zvenigorod	Hoge Veluwe	Liesbos	Oosterhout	Boshoek	Peerdbos	Wytham Woods	Rouvière	Valencia
Va(Laying date)	6.78 [3.45:8.16]	3.72 [1.87:6.85]	4.36 [3.10:6.00]	5.07 [2.70:8.27]	6.53 [4.72:8.73]	4.72 [3.17:8.59]	7.61 [3.60:10.43]	5.89 [4.45:7.84]	7.26 [3.79:13.21]	10.82 [6.19:17.68]
Va(Clutch Size)	0.29 [0.13:0.48]	0.34 [0.13:0.63]	0.64 [0.41:0.86]	0.65 [0.28:0.99]	0.68 [0.41:0.94]	0.62 [0.39:0.93]	0.75 [0.36:0.93]	0.73 [0.53:0.85]	0.66 [0.22:1.65]	0.38 [0.19:0.77]
Va(Fledged)	0.32 [0.20:0.56]	0.85 [0.30:1.47]	0.56 [0.36:0.87]	0.57 [0.31:0.97]	0.65 [0.38:1.05]	0.74 [0.43:1.03]	0.72 [0.36:0.95]	0.50 [0.36:0.69]	0.61 [0.32:1.36]	0.40 [0.18:0.94]
Cov(Laying date,Clutch Size)	0.12 [-0.47:0.61]	-0.11 [-0.74:0.44]	-0.24 [-0.57:0.20]	-0.24 [-0.92:0.58]	0.04 [-0.43:0.57]	-0.46 [-1.12:0.23]	-0.58 [-1.32:0.22]	-0.65 [-1.09:-0.31]	0.02 [-1.59:1.17]	-0.28 [-1.55:0.49]
Cov(Laying date,Fledged)	0.22 [-0.29:0.76]	0.34 [-0.72:1.09]	-0.03 [-0.50:0.38]	-0.02 [-0.88:0.63]	0.06 [-0.59:0.54]	-0.17 [-1.06:0.29]	-0.33 [-1.20:0.32]	-0.54 [-0.87:-0.09]	-0.36 [-1.63:0.84]	-0.07 [-1.72:0.73]
Cov(Clutch Size,Fledged)	0.01 [-0.12:0.16]	0.15 [-0.12:0.51]	0.38 [0.17:0.60]	0.34 [0.14:0.77]	0.65 [0.32:0.89]	0.57 [0.31:0.86]	0.61 [0.28:0.83]	0.50 [0.32:0.63]	0.06 [-0.24:0.86]	0.09 [-0.07:0.48]
Correl(Laying date,Clutch Size)	0.06 [-0.38:0.44]	-0.19 [-0.51:0.43]	-0.08 [-0.32:0.13]	-0.16 [-0.49:0.33]	0.02 [-0.20:0.29]	-0.26 [-0.50:0.12]	-0.23 [-0.57:0.10]	-0.31 [-0.50:-0.18]	0.01 [-0.56:0.40]	-0.28 [-0.55:0.29]
Correl(Laying date,Fledged)	0.15 [-0.19:0.49]	0.20 [-0.40:0.52]	-0.02 [-0.31:0.21]	-0.01 [-0.44:0.39]	-0.08 [-0.24:0.30]	-0.20 [-0.47:0.15]	-0.31 [-0.51:0.21]	-0.29 [-0.48:-0.09]	-0.23 [-0.54:0.35]	-0.27 [-0.65:0.30]
Correl(Clutch Size,Fledged)	0.13 [-0.34:0.44]	0.31 [-0.16:0.74]	0.64 [0.44:0.80]	0.74 [0.53:0.89]	0.88 [0.81:0.94]	0.87 [0.76:0.92]	0.86 [0.74:0.91]	0.81 [0.72:0.87]	0.36 [-0.31:0.73]	0.50 [-0.15:0.75]

Table S12: Unstandardised G-matrix for morphological traits. Va, Cov and Correl respectively correspond to additive genetic variance, genetic covariance and genetic correlation. Values are posterior modes with their 95% credible intervals. Morphological measurements were not available in the Wytham Woods population.

Unstandardized values	Oulu	Zvenigorod	Hoge Veluwe	Liesbos	Oosterhout	Boshoek	Peerdbos	Rouvière	Valencia
Va(tars)	0.11 [0.07:0.16]	0.18 [0.11:0.22]	0.15 [0.10:0.19]	0.11 [0.04:0.18]	0.16 [0.11:0.20]	0.12 [0.10:0.15]	0.09 [0.05:0.11]	0.15 [0.09:0.24]	0.15 [0.10:0.18]
Va(wing)	0.90 [0.68:1.20]	0.70 [0.49:0.92]	0.90 [0.57:1.16]	0.85 [0.39:1.44]	0.54 [0.28:0.79]	0.96 [0.85:1.15]	0.96 [0.83:1.22]	0.70 [0.29:1.04]	1.05 [0.75:1.29]
Va(mass)	0.23 [0.16:0.31]	0.37 [0.22:0.57]	0.31 [0.25:0.38]	0.27 [0.13:0.35]	0.26 [0.14:0.32]	0.30 [0.25:0.34]	0.32 [0.25:0.40]	0.26 [0.13:0.40]	0.28 [0.21:0.37]
Cov(tars,wing)	0.05 [-0.03:0.14]	0.13 [0.05:0.21]	0.06 [-0.04:0.15]	0.18 [-0.05:0.26]	0.05 [-0.04:0.14]	0.08 [0.04:0.13]	0.15 [0.08:0.19]	0.07 [-0.04:0.22]	0.13 [0.05:0.21]
Cov(tars,mass)	0.08 [0.05:0.15]	0.10 [0.03:0.18]	0.12 [0.07:0.16]	0.10 [-0.01:0.14]	0.11 [0.04:0.14]	0.08 [0.06:0.11]	0.06 [0.03:0.10]	0.08 [-0.01:0.15]	0.11 [0.06:0.15]
Cov(wing,mass)	0.14 [0.00:0.22]	0.28 [0.13:0.44]	0.15 [0.08:0.31]	0.20 [0.03:0.44]	0.14 [0.01:0.25]	0.17 [0.11:0.23]	0.30 [0.19:0.38]	0.11 [-0.04:0.33]	0.19 [0.10:0.31]
Correl(tars,wing)	0.17 [-0.08:0.41]	0.43 [0.17:0.60]	0.19 [-0.15:0.41]	0.53 [-0.12:0.76]	0.21 [-0.14:0.43]	0.21 [0.12:0.35]	0.48 [0.32:0.63]	0.36 [-0.15:0.57]	0.32 [0.13:0.50]
Correl(tars,mass)	0.60 [0.39:0.78]	0.47 [0.13:0.67]	0.58 [0.40:0.71]	0.52 [0.10:0.78]	0.51 [0.26:0.67]	0.41 [0.33:0.53]	0.41 [0.24:0.56]	0.34 [-0.02:0.66]	0.55 [0.39:0.67]
Correl(wing,mass)	0.26 [0.04:0.46]	0.58 [0.31:0.74]	0.40 [0.18:0.59]	0.54 [0.10:0.75]	0.35 [0.07:0.63]	0.31 [0.21:0.40]	0.52 [0.39:0.63]	0.49 [-0.03:0.71]	0.41 [0.21:0.54]

Table S13: Standardised G-matrix for life-history traits. G-matrix is standardized by the inter-population mean of the additive genetic variance. Genetic covariances were divided by the square root of the product of mean traits additive genetic variances to preserve the genetic correlation among traits. Va, Cov and Correl respectively correspond to additive genetic variance, genetic covariance and genetic correlation. Values are posterior modes with their 95% credible intervals.

Standardized values	Oulu	Zvenigorod	Hoge Veluwe	Liesbos	Oosterhout	Boshoek	Peerdbos	Wytham woods	Rouvière	Valencia
Va(Laying date)	1.03 [0.53:1.25]	0.57 [0.28:1.05]	0.67 [0.47:0.92]	0.77 [0.41:1.26]	1.00 [0.72:1.33]	0.72 [0.48:1.31]	1.16 [0.55:1.59]	0.90 [0.68:1.20]	1.11 [0.58:2.02]	1.65 [0.94:2.70]
Va(Clutch Size)	0.48 [0.21:0.79]	0.55 [0.21:1.04]	1.06 [0.67:1.41]	1.07 [0.46:1.63]	1.12 [0.68:1.54]	1.01 [0.64:1.54]	1.23 [0.59:1.52]	1.19 [0.88:1.39]	1.08 [0.35:2.71]	0.63 [0.31:1.26]
Va(Fledged)	0.49 [0.31:0.86]	1.29 [0.46:2.25]	0.85 [0.54:1.33]	0.88 [0.47:1.48]	0.98 [0.58:1.59]	1.13 [0.65:1.56]	1.09 [0.55:1.45]	0.76 [0.55:1.06]	0.94 [0.49:2.08]	0.61 [0.28:1.43]
Cov(Laying date,Clutch Size)	0.06 [-0.23:0.30]	-0.05 [-0.37:0.22]	-0.12 [-0.28:0.10]	-0.12 [-0.46:0.29]	0.02 [-0.22:0.29]	-0.23 [-0.56:0.11]	-0.29 [-0.66:0.11]	-0.33 [-0.55:-0.16]	0.01 [-0.80:0.59]	-0.14 [-0.78:0.25]
Cov(Laying date,Fledged)	0.11 [-0.14:0.37]	0.16 [-0.35:0.52]	-0.02 [-0.24:0.19]	-0.01 [-0.43:0.31]	0.03 [-0.28:0.26]	-0.08 [-0.51:0.14]	-0.16 [-0.58:0.16]	-0.26 [-0.42:-0.04]	-0.17 [-0.79:0.40]	-0.03 [-0.83:0.35]
Cov(Clutch Size,Fledged)	0.02 [-0.20:0.25]	0.24 [-0.19:0.81]	0.59 [0.26:0.95]	0.54 [0.21:1.22]	1.03 [0.50:1.40]	0.90 [0.50:1.36]	0.96 [0.45:1.31]	0.79 [0.51:1.00]	0.10 [-0.38:1.35]	0.15 [-0.11:0.77]
Correl(Laying date,Clutch Size)	0.06 [-0.38:0.44]	-0.19 [-0.51:0.43]	-0.08 [-0.32:0.13]	-0.16 [-0.49:0.33]	0.02 [-0.20:0.29]	-0.26 [-0.50:0.12]	-0.23 [-0.57:0.10]	-0.31 [-0.50:-0.18]	0.01 [-0.56:0.40]	-0.28 [-0.55:0.29]
Correl(Laying date,Fledged)	0.15 [-0.19:0.49]	0.20 [-0.40:0.52]	-0.02 [-0.31:0.21]	-0.01 [-0.44:0.39]	-0.08 [-0.24:0.30]	-0.20 [-0.47:0.15]	-0.31 [-0.51:0.21]	-0.29 [-0.48:-0.09]	-0.23 [-0.54:0.35]	-0.27 [-0.65:0.30]
Correl(Clutch Size,Fledged)	0.13 [-0.34:0.44]	0.31 [-0.16:0.74]	0.64 [0.44:0.80]	0.74 [0.53:0.89]	0.88 [0.81:0.94]	0.87 [0.76:0.92]	0.86 [0.74:0.91]	0.81 [0.72:0.87]	0.36 [-0.31:0.73]	0.50 [-0.15:0.75]

Table S14: Standardised G-matrix for morphological traits. G-matrix is standardized by the inter-population mean of the additive genetic variance. Va, Cov and Correl respectively correspond to additive genetic variance, genetic covariance and genetic correlation. Genetic covariances were divided by the square root of the product of mean traits additive genetic variances to preserve the genetic correlation among traits. Values are posterior modes with their 95% credible intervals. Morphological measurements were not available in the Wytham Woods population.

Standardized values	Oulu	Zvenigorod	Hoge Veluwe	Liesbos	Oosterhout	Boshoek	Peerdbos	Rouvière	Valencia
Va(tars)	0.79 [0.51:1.20]	1.35 [0.81:1.59]	1.10 [0.74:1.39]	0.83 [0.33:1.30]	1.13 [0.83:1.47]	0.88 [0.76:1.11]	0.63 [0.38:0.83]	1.13 [0.64:1.76]	1.07 [0.74:1.35]
Va(wing)	1.06 [0.80:1.42]	0.83 [0.57:1.09]	1.06 [0.68:1.37]	1.01 [0.46:1.70]	0.64 [0.33:0.93]	1.14 [1.01:1.36]	1.14 [0.98:1.44]	0.83 [0.34:1.23]	1.24 [0.88:1.53]
Va(mass)	0.78 [0.53:1.07]	1.27 [0.74:1.95]	1.05 [0.87:1.30]	0.93 [0.45:1.22]	0.89 [0.47:1.09]	1.01 [0.85:1.15]	1.11 [0.85:1.37]	0.89 [0.44:1.37]	0.98 [0.71:1.26]
Cov(tars,wing)	0.15 [-0.10:0.40]	0.38 [0.14:0.61]	0.19 [-0.13:0.45]	0.53 [-0.15:0.78]	0.15 [-0.11:0.40]	0.22 [0.13:0.39]	0.44 [0.22:0.55]	0.20 [-0.12:0.63]	0.38 [0.13:0.60]
Cov(tars,mass)	0.41 [0.23:0.73]	0.50 [0.15:0.89]	0.58 [0.37:0.80]	0.52 [-0.03:0.71]	0.54 [0.20:0.70]	0.40 [0.30:0.54]	0.32 [0.17:0.52]	0.38 [-0.05:0.76]	0.55 [0.31:0.76]
Cov(wing,mass)	0.27 [0.01:0.45]	0.55 [0.26:0.89]	0.31 [0.15:0.62]	0.41 [0.06:0.88]	0.28 [0.02:0.51]	0.34 [0.23:0.46]	0.60 [0.38:0.76]	0.22 [-0.07:0.67]	0.39 [0.19:0.63]
Correl(tars,wing)	0.17 [-0.08:0.41]	0.43 [0.17:0.60]	0.19 [-0.15:0.41]	0.53 [-0.12:0.76]	0.21 [-0.14:0.43]	0.21 [0.12:0.35]	0.48 [0.32:0.63]	0.36 [-0.15:0.57]	0.32 [0.13:0.50]
Correl(tars,mass)	0.60 [0.39:0.78]	0.47 [0.13:0.67]	0.58 [0.40:0.71]	0.52 [0.10:0.78]	0.51 [0.26:0.67]	0.41 [0.33:0.53]	0.41 [0.24:0.56]	0.34 [-0.02:0.66]	0.55 [0.39:0.67]
Correl(wing,mass)	0.26 [0.04:0.46]	0.58 [0.31:0.74]	0.40 [0.18:0.59]	0.54 [0.10:0.75]	0.35 [0.07:0.63]	0.31 [0.21:0.40]	0.52 [0.39:0.63]	0.49 [-0.03:0.71]	0.41 [0.21:0.54]

Table S15: P-matrix for life-history traits. Vp, Cov and Correl respectively correspond to phenotypic variance, genetic covariance and genetic correlation. P-matrix corresponds to the sum random parameters inferred with the animal models. Values are posterior modes with their 95% credible intervals.

Standardized values	Oulu	Zvenigorod	Hoge Veluwe	Liesbos	Oosterhout	Boshoek	Peerdbos	Wytham Woods	Rouvière	Valencia
Vp(Laying date)	37.89 [30.51:49.64]	31.15 [24.67:40.67]	58.75 [50.15:74.77]	57.23 [47.93:74.47]	57.91 [48.61:80.61]	64.59 [54.21:99.64]	84.07 [68.76:109.09]	71.53 [59.37:93.40]	75.33 [57.40:106.48]	56.18 [48.22:74.66]
Vp(Clutch Size)	2.56 [2.31:2.91]	2.01 [1.76:2.40]	3.37 [3.14:3.66]	3.50 [3.18:3.91]	2.46 [2.30:2.75]	2.60 [2.42:2.85]	2.71 [2.44:3.17]	2.69 [2.54:2.96]	4.17 [3.72:4.87]	2.04 [1.79:2.46]
Vp(Fledged)	5.74 [5.16:6.91]	5.94 [5.01:7.12]	6.92 [6.48:7.65]	5.33 [4.74:5.89]	3.70 [3.47:4.10]	4.33 [3.97:4.76]	4.81 [4.32:5.61]	5.01 [4.66:5.41]	6.72 [5.68:8.02]	3.57 [2.99:5.31]
Cov(Laying date,Clutch Size)	-0.60 [-2.01:0.48]	-0.95 [-2.19:0.13]	-2.46 [-3.39:-0.80]	-0.63 [-2.27:0.76]	-0.31 [-1.30:1.18]	-2.91 [-4.62:-1.58]	-2.01 [-4.06:-0.33]	-3.17 [-4.61:-1.83]	-0.90 [-2.96:1.54]	-1.79 [-3.44:-0.31]
Cov(Laying date,Fledged)	-1.68 [-3.75:0.53]	0.08 [-2.28:2.04]	-2.25 [-4.39:-0.47]	-0.02 [-1.81:2.02]	-0.18 [-1.41:1.50]	-3.25 [-5.49:-1.13]	-2.28 [-4.79:0.45]	-3.76 [-5.59:-2.09]	-0.01 [-4.14:2.88]	-2.46 [-6.15:1.53]
Cov(Clutch Size,Fledged)	1.33 [1.03:1.89]	1.40 [1.07:2.01]	2.22 [1.94:2.56]	2.96 [2.56:3.37]	2.28 [2.08:2.55]	2.08 [1.87:2.37]	2.42 [2.11:2.94]	2.24 [2.00:2.50]	2.10 [1.30:2.64]	0.94 [0.28:1.48]
Correl(Laying date,Clutch Size)	-0.04 [-0.20:0.04]	-0.15 [-0.28:0.00]	-0.18 [-0.23:-0.06]	-0.05 [-0.15:0.05]	-0.03 [-0.11:0.08]	-0.23 [-0.31:-0.11]	-0.13 [-0.25:-0.02]	-0.24 [-0.31:-0.14]	-0.04 [-0.16:0.08]	-0.18 [-0.30:-0.03]
Correl(Laying date,Fledged)	-0.14 [-0.23:0.02]	0.01 [-0.17:0.13]	-0.13 [-0.21:-0.03]	0.00 [-0.09:0.12]	0.01 [-0.09:0.09]	-0.20 [-0.31:-0.09]	-0.12 [-0.23:0.02]	-0.19 [-0.28:-0.11]	0.00 [-0.18:0.12]	-0.19 [-0.35:0.10]
Correl(Clutch Size,Fledged)	0.36 [0.29:0.44]	0.44 [0.34:0.53]	0.45 [0.42:0.50]	0.68 [0.64:0.71]	0.76 [0.73:0.78]	0.64 [0.59:0.67]	0.68 [0.64:0.73]	0.60 [0.57:0.63]	0.38 [0.25:0.47]	0.37 [0.12:0.49]

Table S16: P-matrix for morphological traits. Vp, Cov and Correl respectively correspond to phenotypic variance, genetic covariance and genetic correlation. P-matrix corresponds to the sum random parameters inferred with the animal models. Values are posterior modes with their 95% credible intervals. Morphological measurements were not available in the Wytham Woods population.

Standardized values	Oulu	Zvenigorod	Hoge Veluwe	Liesbos	Oosterhout	Boshoek	Peerdbos	Rouvière	Valencia
Vp(tars)	0.42 [0.39:0.46]	0.42 [0.38:0.47]	0.37 [0.35:0.39]	0.61 [0.55:0.84]	0.39 [0.36:0.43]	0.34 [0.32:0.37]	0.35 [0.33:0.39]	0.35 [0.30:0.38]	0.37 [0.36:0.42]
Vp(wing)	2.86 [2.64:3.07]	2.44 [2.32:2.73]	3.05 [2.85:3.35]	5.16 [4.13:7.38]	3.57 [3.12:4.35]	2.65 [2.51:2.91]	2.28 [2.16:2.50]	3.48 [3.18:4.00]	3.62 [3.23:4.20]
Vp(mass)	1.01 [0.97:1.17]	1.58 [1.43:1.72]	0.90 [0.87:0.93]	1.21 [1.10:1.49]	0.99 [0.94:1.10]	0.60 [0.57:0.65]	0.73 [0.68:0.79]	0.83 [0.73:0.91]	0.86 [0.81:0.95]
Cov(tars,wing)	0.23 [0.17:0.29]	0.19 [0.12:0.27]	0.13 [0.09:0.19]	0.01 [-0.50:0.64]	0.17 [0.06:0.28]	0.11 [0.05:0.16]	0.18 [0.13:0.23]	0.22 [0.13:0.31]	0.21 [0.12:0.29]
Cov(tars,mass)	0.18 [0.15:0.24]	0.13 [0.06:0.19]	0.18 [0.16:0.19]	0.14 [0.03:0.25]	0.13 [0.07:0.17]	0.12 [0.09:0.14]	0.12 [0.09:0.15]	0.16 [0.12:0.22]	0.21 [0.18:0.25]
Cov(wing,mass)	0.42 [0.30:0.53]	0.40 [0.24:0.51]	0.29 [0.22:0.36]	0.49 [0.16:0.93]	0.42 [0.21:0.63]	0.25 [0.20:0.33]	0.37 [0.31:0.45]	0.42 [0.26:0.57]	0.40 [0.26:0.53]
Correl(tars,wing)	0.21 [0.16:0.26]	0.18 [0.11:0.25]	0.13 [0.09:0.18]	0.00 [-0.25:0.30]	0.14 [0.05:0.22]	0.11 [0.04:0.16]	0.22 [0.14:0.24]	0.19 [0.10:0.26]	0.17 [0.10:0.24]
Correl(tars,mass)	0.27 [0.22:0.35]	0.17 [0.08:0.23]	0.31 [0.28:0.33]	0.18 [0.04:0.26]	0.20 [0.12:0.28]	0.26 [0.20:0.30]	0.25 [0.17:0.29]	0.34 [0.24:0.39]	0.36 [0.32:0.42]
Correl(wing,mass)	0.25 [0.18:0.30]	0.19 [0.13:0.26]	0.18 [0.13:0.21]	0.21 [0.06:0.32]	0.24 [0.12:0.32]	0.20 [0.15:0.25]	0.28 [0.24:0.34]	0.24 [0.17:0.33]	0.22 [0.14:0.30]

Table S17: Estimates of heritability (posterior mode and 95%Credible Interval). P-matrices were calculated as the sum of all the variance– covariance matrices associated with the different random effects. Heritabilities were estimated as the product $P^{-1/2}GP^{-1/2}$ by using the unstandardized G-matrix and P-matrix.

Population	Clutch size	Laying date	Number of fledged	Body mass	Tarsus length	Wing length
Oulu	0.13[0.06:0.21]	0.16[0.08:0.22]	0.06[0.04:0.11]	0.19[0.14:0.27]	0.26[0.14:0.35]	0.32[0.25:0.42]
Zvenigorod	0.17[0.08:0.32]	0.11[0.05:0.21]	0.15[0.07:0.26]	0.23[0.12:0.32]	0.38[0.24:0.48]	0.25[0.18:0.34]
Hoge Veluwe	0.19[0.13:0.25]	0.07[0.05:0.11]	0.08[0.04:0.10]	0.33[0.25:0.38]	0.37[0.24:0.47]	0.30[0.18:0.36]
Liesbos	0.16[0.09:0.27]	0.08[0.04:0.14]	0.11[0.06:0.15]	0.19[0.10:0.26]	0.18[0.06:0.26]	0.14[0.06:0.27]
Oosterhout	0.27[0.16:0.32]	0.11[0.06:0.14]	0.13[0.08:0.20]	0.23[0.13:0.29]	0.39[0.29:0.49]	0.14[0.07:0.21]
Boshoek	0.22[0.14:0.30]	0.07[0.04:0.13]	0.13[0.08:0.17]	0.46[0.40:0.52]	0.32[0.27:0.39]	0.36[0.31:0.40]
Peerdbos	0.24[0.15:0.32]	0.08[0.04:0.13]	0.10[0.05:0.13]	0.39[0.31:0.48]	0.20[0.12:0.27]	0.40[0.34:0.48]
Wytham Woods	0.25[0.20:0.30]	0.07[0.05:0.11]	0.06[0.05:0.09]	**	**	**
Rouvière	0.13[0.06:0.38]	0.10[0.05:0.18]	0.10[0.05:0.19]	0.29[0.18:0.45]	0.51[0.27:0.65]	0.18[0.08:0.27]
Valencia	0.20[0.09:0.35]	0.18[0.09:0.29]	0.13[0.05:0.24]	0.29[0.22:0.37]	0.34[0.25:0.43]	0.28[0.20:0.34]

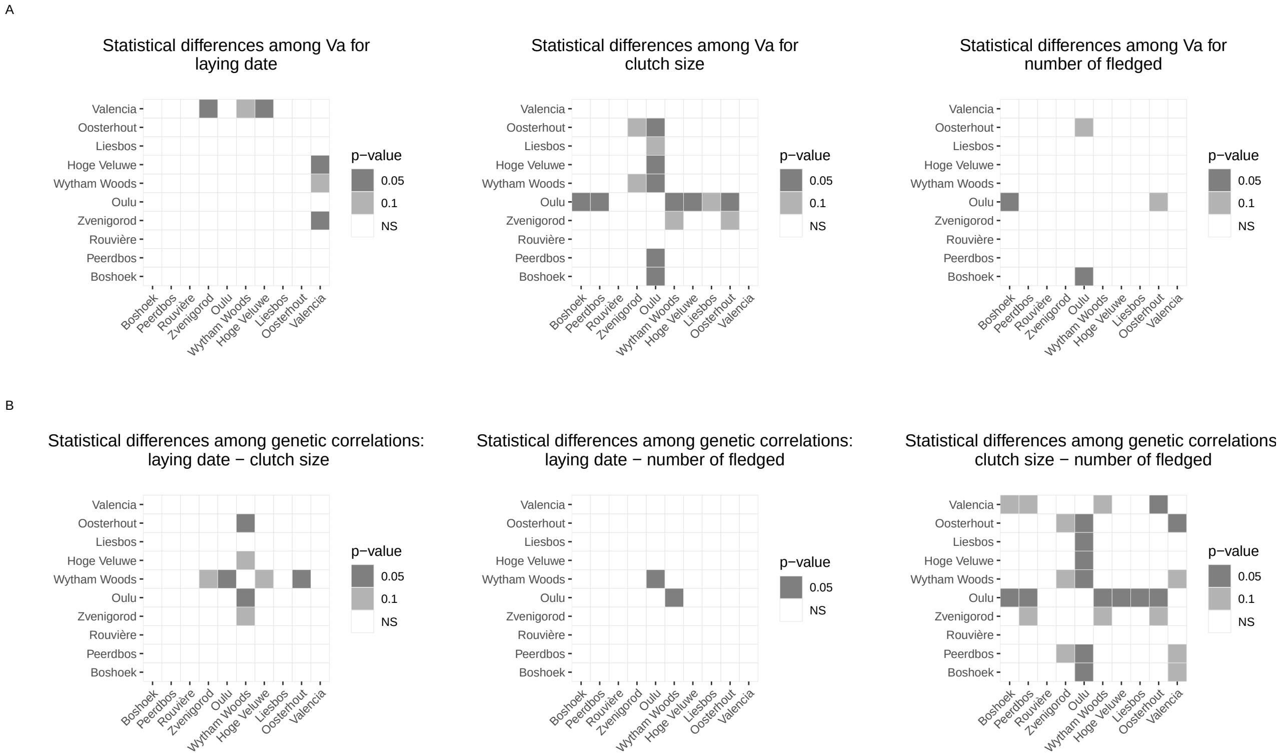

Figure S1: Statistical differences between (A) additive genetic values and (B) genetic correlations for life-history traits. To estimate the statistical differences, we calculated the pairwise differences between populations for each genetic variance and correlation based on 1000 posterior samples of the Bayesian animal model outputs. Then, we estimated the 95% and 90% credible interval on these pairwise differences and concluded that there was a statistically significant difference at a respective p-value of 0.05 (dark grey) and 0.1 (light grey) when the credible interval did not cross 0.

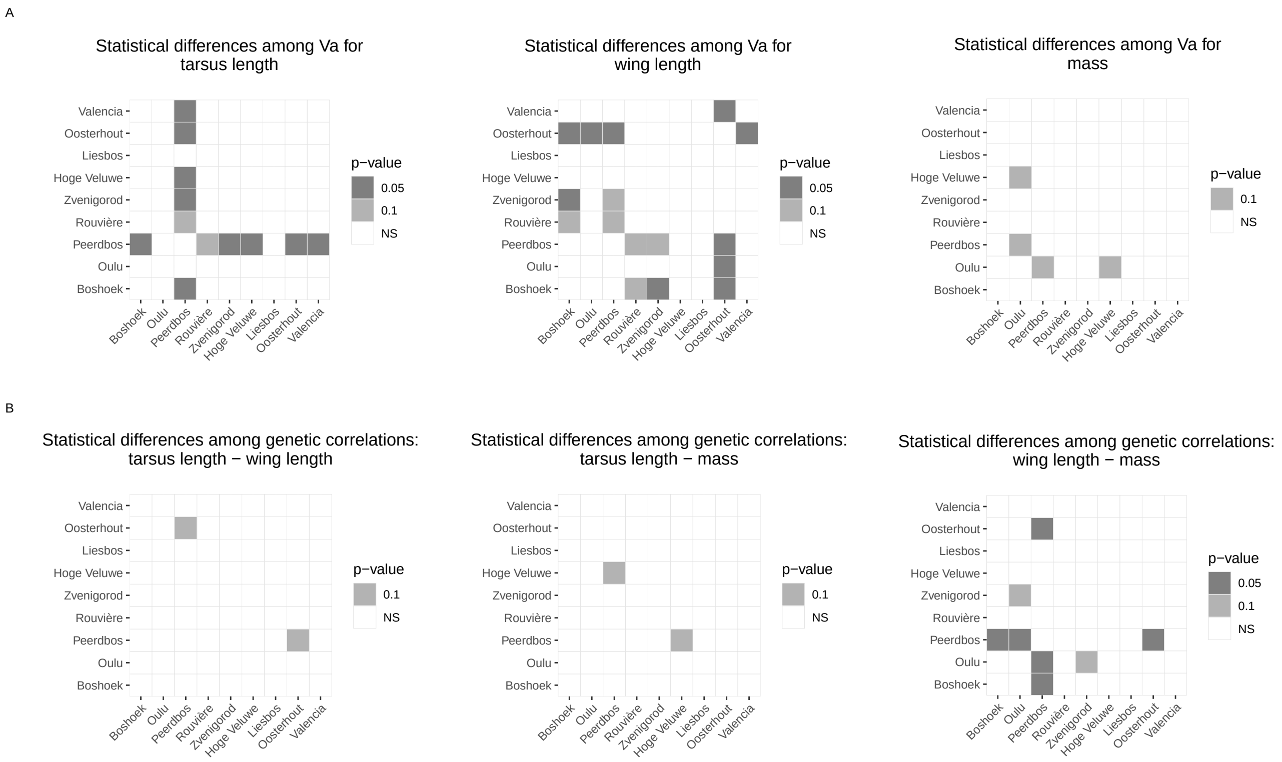

Figure S2: Statistical differences between (A) additive genetic values and (B) genetic correlations for life morphological traits. To estimate the statistical differences, we calculated the pairwise differences between populations for each genetic variance and correlation based on 1000 posterior samples of the Bayesian animal model outputs. Then, we estimated the 95% and 90% credible interval on these pairwise differences and concluded that there was a statistically significant difference at a respective p-value of 0.05 (dark grey) and 0.1 light (grey) when the credible interval did not cross 0.

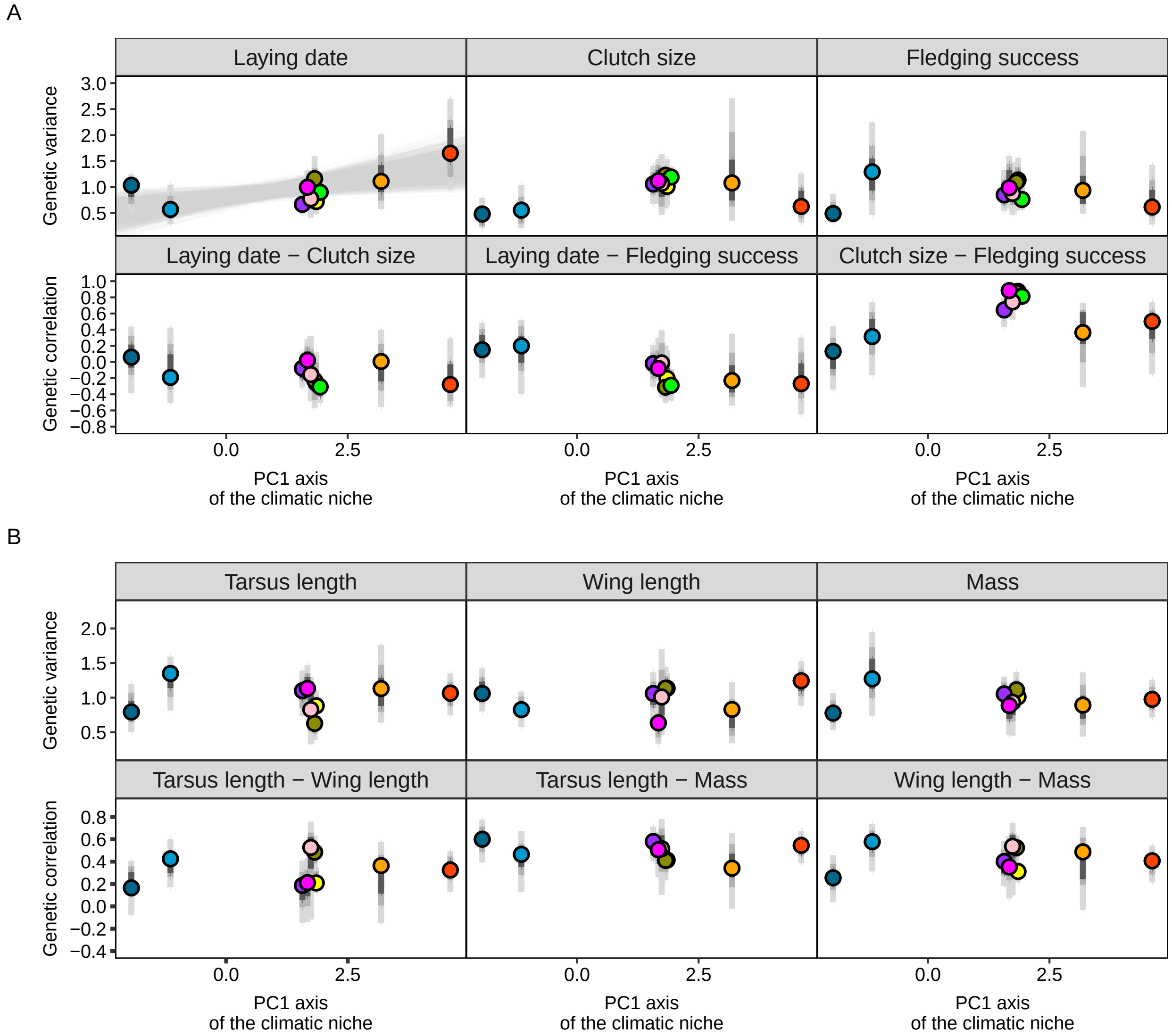

Figure S3: Standardized additive genetic variances and genetic correlations estimated for (A) life-history and (B) morphological traits, plotted along the PC1 axis of the climatic niche. Points represents the posterior mode estimates filled with colors corresponding to their populations (see main text Fig.1) and grey bars the 95%, 75% and 50% credible intervals (respectively light grey, medium grey and dark grey).

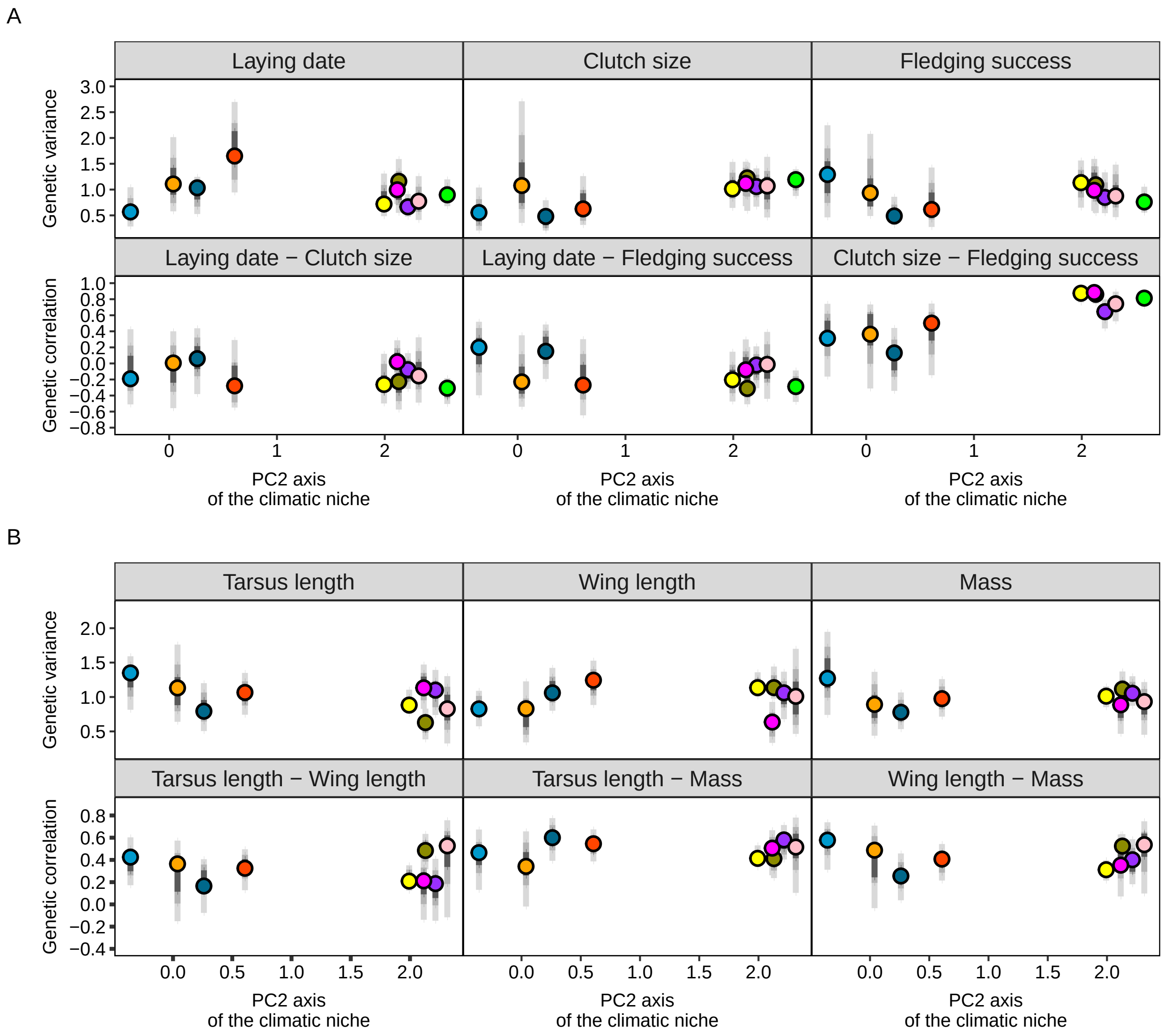

Figure S4: Standardized additive genetic variances and genetic correlations estimated for (A) life-history and (B) morphological traits, plotted along the PC2 axis of the climatic niche. Points represents the posterior mode estimates filled with colors corresponding to their populations (see main text Fig.1) and grey bars the 95%, 75% and 50% credible intervals (respectively light grey, medium grey and dark grey).

Supporting Information 5: Details on tensor method and results

The variation in genetic covariance structure across populations was assessed using genetic covariance tensors (Hine et al. 2009). Instead of making pairwise comparisons between G-matrices, the tensor method studies changes in the structure of G-matrix along a gradient. Indeed, the tensor approach describes the variation between G-matrices based on successive linear transformations and identifies independent combinations of traits that have undergone a change in genetic variance. A full description of the theoretical aspect the genetic covariance tensor can be found in Hine et al. (2009).

Briefly, tensor is mathematical object which can be represented as multidimensional array with certain transformation properties. The order of a tensor reflects the number of indices required to define elements in a vectorial space. Using this definition, a G-matrix can be considered as second-order tensor variables as two indices are necessary to refer to the covariance between two traits x and y, e.g. G_{xy}. The variation among G-matrices of several populations can be characterized by a fourth- order genetic covariance tensor. Indeed, four indices are required to refer to the between population covariance of trait covariance, e.g. $G_{xy,wz}$ refer to the covariance between G_{xy} and G_{wz}.

In practice, the genetic covariance tensor is represented by a matrix (**S**) of dimension n(n+1)/2, (where n is the number of traits), which contains the variances and covariances among the elements of the G-matrices. **S** can be decomposed into k eigenvectors and their associated eigenvalues. Each eigenvector represents an independent change in the variance-covariance structure among G-matrices, and its respective eigenvalues represent the amount of variation among G-matrices explained by the eigenvector. These eigenvector of **S** can be rearranged in matrix form to give the respective eigentensor (E^k). Then, the eigentensors can be decomposed into p eigenvectors ($v_{p,k}$) and eigenvalues ($e_{p,k}$) to assess which independent linear combinations of traits have contributed to the change in genetic variance in the space of eigentensors. As the G-matrices can be described as a linear combination of the eigentensors, it becomes possible to estimate the coordinates of a G-matrix on each eigentensor by solving the following equation:

$$G_j = \sum_{k=1}^{n} C^{i,j} E^k$$

where G_j is the G-matrix for population j, n is the maximum number of non-zero eigentensors, E^k is the kth eigentensor, and $C^{i,j}$ is the coordinate for the ith estimates of the jth population. The coordinates offer the opportunity to describe how much the populations differ along a given tensor. In other words, when two G-matrices are close with respect to a particular tensor, then these matrices are described in the same way by the eigentensor.

We applied this method on the posterior estimates of the animal models following the Aguirre et al. (2013). Indeed, we computed the $\mathbf{S}_i$ matrix for each of the ith samples (here 1000) of our posterior distribution of G-matrices. Then, we calculated the posterior mean $\overline{S}$ of the elements of $\mathbf{S}_i$ and projected every i^{th} sample of G-matrix on the different eigentensors $\overline{E_k}$ (estimated on $\overline{S}$) to obtain the distribution of the coordinates of the different G-matrices on each eigentensor.

We tested the results against a null model. Generation of null G-matrix are detailed in the manuscript. Then for both datasets (observed G-matrix and null G-matrix), we projected the kth eigenvector of onto $\mathbf{S}_i$ in order to determine α_{ik} , the variance among the ith MCMC sample of the G-matrices for the aspect of covariance structure specified by $\overline{E_k}$. The posterior distribution of α_{ik} summarizes the uncertainty in the variance in the covariance structure represented by $\overline{E_k}$. To test whether the eigentensors describe significant variation among the observed G-matrices, we compared the α_{ik} estimated on observed and null G-matrix.

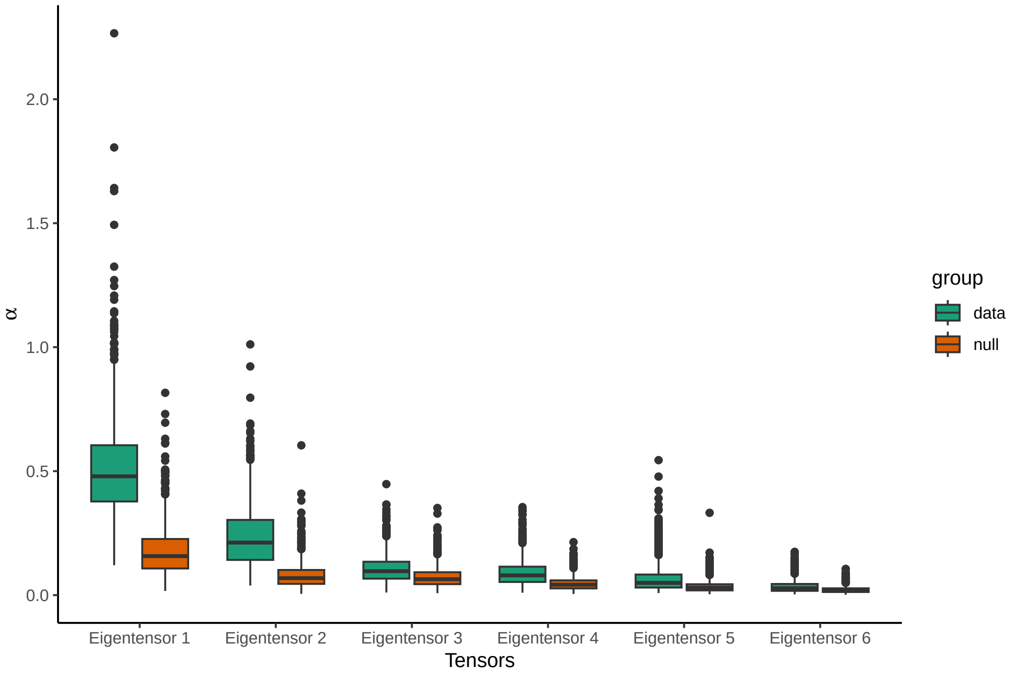

Figure S5: Variance accounted for by each eigentensor (α) for the observed G-matrices of life-history traits (green) and the null G-matrices (orange). See description above for details.

Table S18: Significant differences between the eigentensors estimated from the observed data and the eigentensors estimated from null model for life-history traits. p-values were estimated as the difference between α(data) and α(null) divided by the number of posterior sample (here 1000).

Eigentensor	p-value for significant α(data)>α(null)
1	0.034
2	0.094
3	0.301
4	0.213
5	0.292
6	0.337

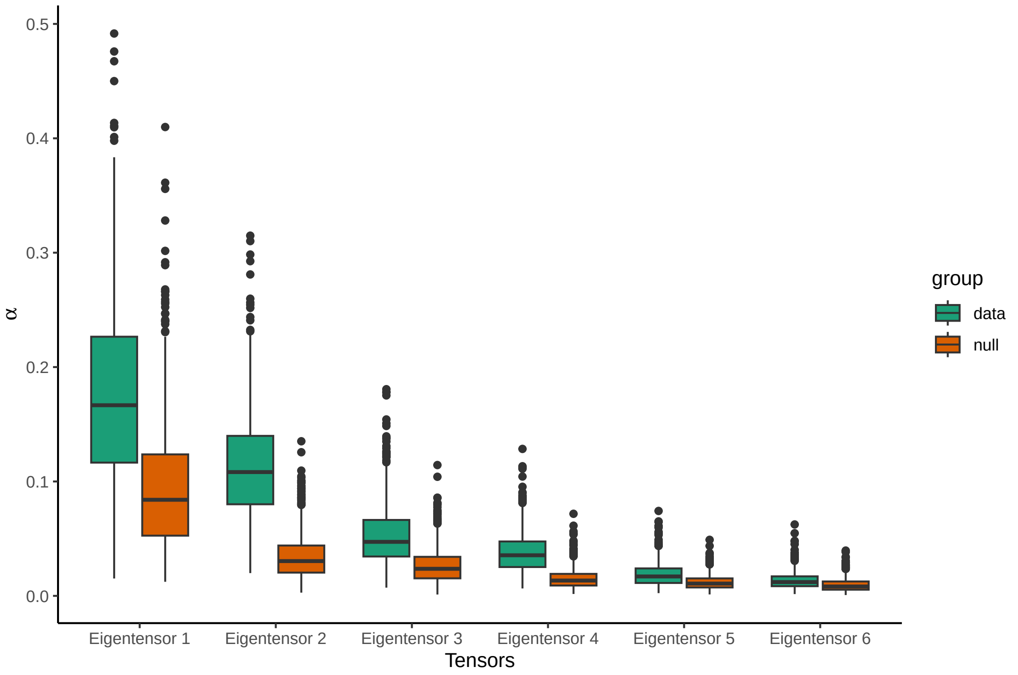

Figure S6: Variance accounted for by each eigentensor (α) for the observed G-matrices of morphological traits (green) and the null G-matrices (orange). See description above for details.

Table S19: Significant differences between the eigentensors estimated from the observed data and the eigentensors estimated from null model for morphological traits. p-values were estimated as the difference between α(data) and α(null) divided by the number of posterior sample (here 1000).

**Eigentensor**	**p-value for significant α(data)>α(null)**
1	0.187
2	0.035
3	0.172
4	0.095
5	0.308
6	0.310

Table S20: Posterior mode of the coordinates of populations with 95% Credible Interval (CI) on the first and second tensors for G-matrices of life-history traits. Modes were estimated using adjust option equal 1.

Population	Coordinate on first tensor [95%CI]	Coordinate on second tensor [95%CI]
Oulu	-0.31[-0.80:-0.13]	0.43[0.08:0.84]
Zvenigorod	-0.85[-1.88:-0.19]	0.09[-0.48:0.67]
Hoge Veluwe	-1.52[-2.12:-0.81]	0.32[-0.02:0.64]
Liesbos	-1.55[-2.65:-0.65]	0.35[-0.24:0.90]
Oosterhout	-1.87[-2.73:-0.92]	0.26[-0.18:0.67]
Boshoek	-2.02[-2.99:-1.15]	0.45[-0.11:0.96]
Peerdbos	-1.99[-2.83:-1.01]	0.53[-0.02:1.31]
Wytham wood	-1.87[-2.34:-1.31]	0.70[0.37:1.00]
Rouvière	-1.01[-3.20:-0.18]	0.75[-0.03:1.82]
Valencia	-0.78[-2.05:-0.23]	1.50[0.40:2.35]

Table S21: Posterior mode of coordinates of populations with 95% Credible Interval (CI) on the first and second tensors for G-matrices of morphological traits. Modes were estimated using adjust option equal 1.

Population	Coordinate on first tensor[95%CI]	Coordinate on second tensor [95%CI]
Oulu	-1.46[-1.93:-0.86]	0.45[0.09:0.89]
Zvenigorod	-2.05[-2.70:-1.28]	0.96[0.39:1.35]
Hoge Veluwe	-1.66[-2.10:-1.08]	0.80[0.38:1.13]
Liesbos	-1.85[-2.62:-0.62]	0.35[-0.13:0.96]
Oosterhout	-1.18[-1.75:-0.65]	0.88[0.54:1.28]
Boshoek	-1.60[-1.88:-1.35]	0.43[0.26:0.63]
Peerdbos	-1.85[-2.31:-1.50]	0.16[-0.09:0.44]
Rouvière	-1.35[-2.20:-0.48]	0.80[0.20:1.38]
Valencia	-1.76[-2.34:-1.32]	0.59[0.23:0.95]

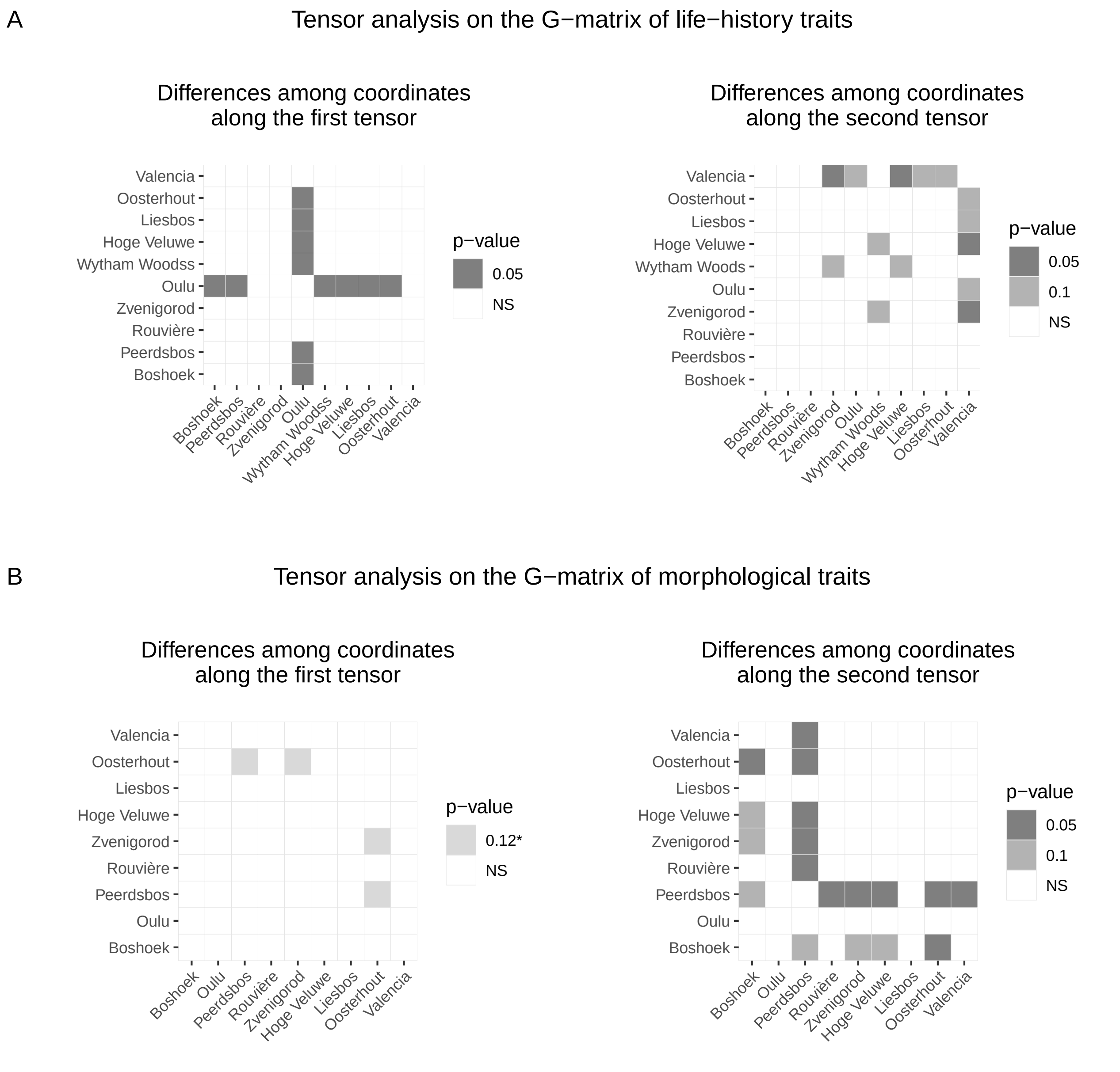

Figure S7: Statistical differences in coordinate values among populations for (A) life-history and (B) morphological traits. Left and right panels represent the two first tensors. To estimate the statistical differences, we calculated the pairwise differences between populations for each coordinate based on the 1000 posterior samples estimated by the genetic covariance tensor analysis. Then, we estimated the 95% and 90% credible interval on these pairwise differences and concluded that there was a statistically significant difference at a respective p-value of 0.05 and 0.1 when the credible interval did not cross 0. *Note that for the tensor analysis of morphological traits, differences among coordinates along the first tensor were significative at a p-value threshold of 0.12.

Table S22: Results of the tensor analysis on standardised G-matrices for life-history traits for the two first eigentensors. The eigentensors k are decomposed into p eigenvectors ($v_{p,k}$), see text SI 5 for details.

**Tensors**	**% variance explained [95%CI]**	**Eigenvectors**	**Eigenvalues**	**Laying Date**	**Clutch Size**	**Fledging success**
tensor1 (k=1)	48 [26:71]	first ($v_{1,1}$)	-0.99	0.23	-0.74	-0.64
		second ($v_{2,1}$)	0.16	0.25	-0.59	0.77
		third ($v_{3,1}$)	0.03	0.94	0.34	-0.05
tensor2 (k=2)	16 [5:41]	first ($v_{1,2}$)	0.90	0.93	-0.27	-0.23
		second ($v_{2,2}$)	-0.42	-0.34	-0.48	-0.81
		third ($v_{3,2}$)	0.13	-0.11	-0.83	0.54

Table S23: Results of the tensor analysis on standardised G-matrices for morphological traits for the two first eigentensors. The eigentensors k are decomposed into p eigenvectors ($v_{p,k}$), see text SI 5 for details.

**Tensor**	**% variance explained [95%CI]**	**Eigenvector**	**Eigenvalue**	**Tarsus**	**Wing**	**Mass**
tensor1 (k=1)	37 [20:65]	first ($v_{1,1}$)	-0.99	-0.41	-0.66	-0.63
		second ($v_{2,1}$)	0.14	0.79	-0.60	0.12
		third ($v_{3,1}$)	-0.07	-0.46	-0.45	0.76
tensor2 (k=2)	24 [11:47]	first ($v_{1,2}$)	0.87	0.91	-0.05	0.41
		second ($v_{2,2}$)	-0.49	0.02	1.00	0.08
		third ($v_{3,2}$)	0.03	-0.41	-0.06	0.91

Results of tensor analyse when the two extreme populations are removed

In order to understand whether the overall pattern for life-history traits is mainly driven by the two extreme populations, we re-run tensor analyse by removing the two extreme populations one by one or both simultaneously. However, note that removing these populations also remove an important part of the gradient and power.

When the Spanish population is removed, the first eigenaxis from the tensor analysis is still significantly different from the null model ($p\text{-value}_1 = 0.037$, $p\text{-value}_2 = 0.21$). Regarding the slope of the regression between tensor distance and niche distance, the relationship is still significant ($slope_{data}$[95%CI]=0.13 [0.06:0.24]), and different from the null expectation ($slope_{null}$[95%CI]=0.02 [-0.06:0.07], $slope_{data}$ is statistically higher than $slope_{null}$ at a p-value of 0.01).

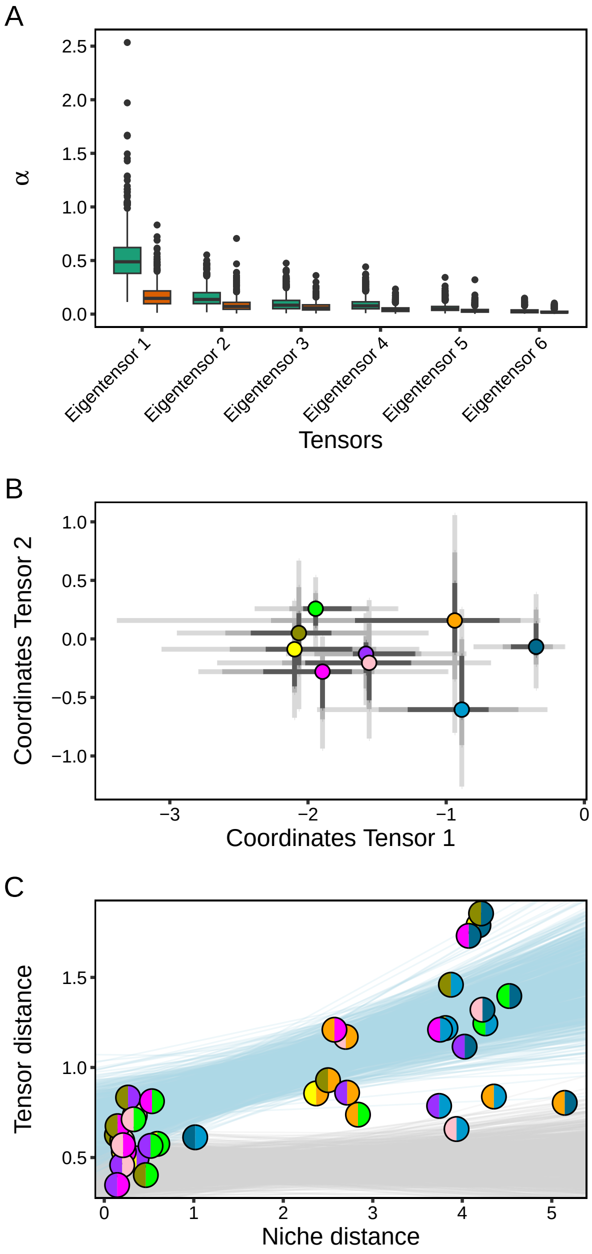

Figure S8: Tensor analysis when the Spanish population is removed. Panels show (A) the null model (B) the coordinates on the two first tensors and (C) regression between tensor distance and niche distance.

When the Finnish population is removed, the two first eigenaxis from the tensor analysis are marginally significantly different from the null model (p-value$_1$=0.144, p-value$_2$=0.096). Regarding the slope of the regression between tensor distance and niche distance, the relationship is still significant (slope$_{data}$[95%CI]=0.137 [0.035:0.219]) and different from the null expectation (slope$_{null}$[95%CI]=0.01 [-0.05:0.094], slope$_{data}$ is statistically higher than slope$_{null}$ at a p-value of 0.04).

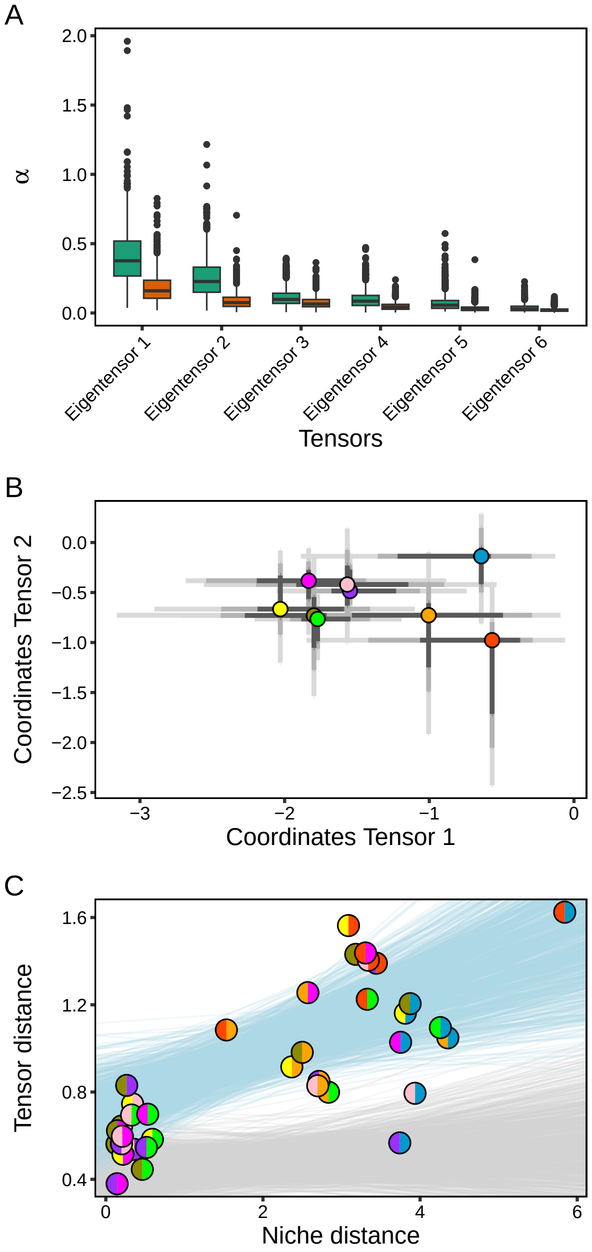

Figure S9: Tensor analysis when the Finnish population is removed. Panels show (A) the null model (B) the coordinates on the two first tensors and (C) regression between tensor distance and niche distance.

When both, the Spanish and the Finish populations are removed, the two first eigentensor are not significant (p-value$_1$=0.15, p-value$_2$=0.23). It should be noted that this removes one fifth of the data and that the sensitivity of the null model to the number of populations used and number of traits is unknown (neither empirically nor analytically). The slope between the niche distance and tensor distance is still significant even when these two extreme populations are removed (slope$_{data}$[95%CI]=0.13 [0.02:0.23]), but unsurprisingly not different from the null model anymore (slope$_{null}$[95%CI]=0.07 [-0.004:0.15], slope$_{data}$ is statistically higher than slope$_{null}$ at a p-value of 0.28).

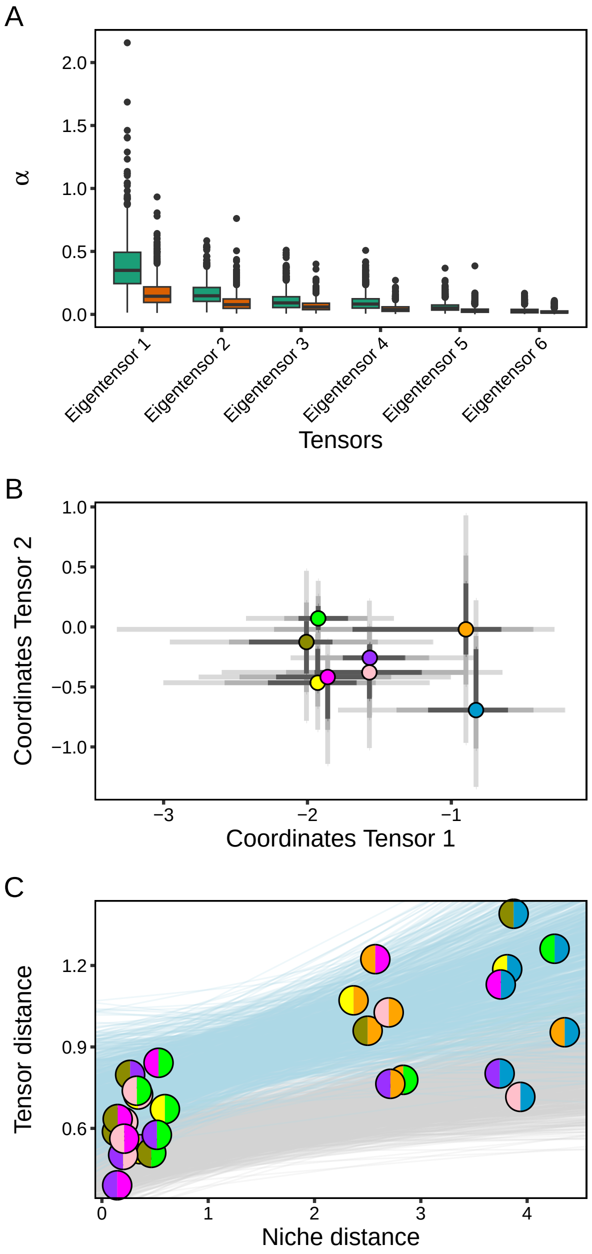

Figure S10: Tensor analysis when both the Finnish and Spanish population are removed. Panels show (A) the null model (B) the coordinates on the two first tensors and (C) regression between tensor distance and niche distance.

Supporting Information 6: Angle analysis details and results

Following Robinson et al. (2013), the statistical support for a significant angle between g_{max} (Θ) of two matrices can be easily calculated as by drawing two random samples (A, B) from the posterior distribution of G-matrix of the population j ($Gpop_j$) and population k ($Gpop_k$), and calculate as:

$$\begin{aligned}\Psi\left(Gpop_d, Gpop_f\right) = & \left[\Theta\left(Gpop_d^A, Gpop_d^B\right) + \Theta\left(Gpop_f^A, Gpop_f^B\right)\right] \\ & - \left[\Theta\left(Gpop_d^A, Gpop_f^A\right) + \Theta\left(Gpop_d^B, Gpop_f^B\right)\right]\end{aligned}$$

The first part of the right-hand side of the equation represents the angle between g_{max} within populations and the second part the angle between populations, it is thus a test of whether the differences between populations are larger than the differences within populations. The statistical support of the difference between two matrices can be evaluated by using all the point estimates of their posterior distributions as random samples and compute the 95% credible interval of Ψ.

Table S24: Angle values between g_{max} for each pair of populations for the G-matrix of life-history traits. Ψ are provided with the 95% Credible Interval (see above for details).

Population 1	Population 2	Angle [95%CI]	Ψ [95%CI]
Boshoek	Peerdbos	6.65 [0.12:42.37]	0.08 [-31.48:30.72]
Boshoek	Rouvière	19.41 [6.88:86.20]	-0.47 [-89.18:31.18]
Boshoek	Zvenigorod	32.06 [5.58:71.66]	-34.98 [-85.37:19.78]
Boshoek	Oulu	85.45 [37.74:89.99]	-96.95 [-150.33:-0.20]
Boshoek	Wytham Woods	11.25 [1.49:31.67]	-2.94 [-39.26:11.60]
Boshoek	Hoge Veluwe	6.44 [1.29:31.26]	0.02 [-32.02:21.21]
Boshoek	Liesbos	7.07 [0.31:60.68]	-0.13 [-49.05:27.62]
Boshoek	Oosterhout	18.08 [0.24:41.82]	-0.09 [-45.92:22.30]
Boshoek	Valencia	58.73 [18.26:89.89]	-55.34 [-133.20:9.73]
Peerdbos	Rouvière	40.20 [7.52:86.88]	0.05 [-82.85:46.06]
Peerdbos	Zvenigorod	28.64 [11.14:80.76]	-31.96 [-99.65:15.55]
Peerdbos	Oulu	84.67 [31.41:89.99]	-100.60 [-151.85:8.99]
Peerdbos	Wytham Woods	8.90 [0.62:34.72]	-0.08 [-31.90:19.36]
Peerdbos	Hoge Veluwe	12.43 [0.92:45.43]	-0.25 [-44.70:23.66]
Peerdbos	Liesbos	9.82 [0.63:63.09]	-0.21 [-62.65:35.67]
Peerdbos	Oosterhout	19.52 [0.87:52.60]	-0.01 [-59.57:26.93]
Peerdbos	Valencia	40.83 [15.75:89.83]	-0.25 [-131.55:14.00]
Rouvière	Zvenigorod	54.99 [17.00:89.66]	-46.90 [-104.03:48.13]
Rouvière	Oulu	88.02 [20.64:89.98]	2.11 [-132.05:49.22]
Rouvière	Wytham Woods	14.26 [4.82:85.14]	-0.31 [-89.92:18.10]
Rouvière	Hoge Veluwe	18.71 [5.77:84.43]	-0.10 [-95.03:29.12]
Rouvière	Liesbos	35.39 [8.50:87.24]	-0.01 [-89.25:59.50]
Rouvière	Oosterhout	32.82 [8.71:88.48]	-0.11 [-100.75:30.67]
Rouvière	Valencia	60.67 [13.68:89.96]	-0.35 [-114.78:55.42]
Zvenigorod	Oulu	61.21 [25.41:89.79]	-56.45 [-134.59:18.32]
Zvenigorod	Wytham Woods	37.13 [13.60:82.28]	-59.23 [-109.50:0.83]
Zvenigorod	Hoge Veluwe	35.93 [8.14:74.38]	-25.03 [-82.69:14.50]
Zvenigorod	Liesbos	35.60 [10.39:82.01]	-35.75 [-85.16:19.08]
Zvenigorod	Oosterhout	22.93 [4.85:65.98]	-33.73 [-70.47:16.25]
Zvenigorod	Valencia	77.66 [28.95:89.94]	-100.41 [-138.45:23.62]
Oulu	Wytham Woods	88.96 [39.53:89.92]	-126.16 [-164.19:-21.11]
Oulu	Hoge Veluwe	84.76 [34.99:90.00]	-114.45 [-150.17:0.81]
Oulu	Liesbos	82.63 [23.34:89.87]	-76.36 [-148.42:20.80]
Oulu	Oosterhout	88.56 [28.36:89.98]	-105.44 [-148.10:2.11]
Oulu	Valencia	31.73 [8.97:85.73]	-0.21 [-114.10:38.09]
Wytham Woods	Hoge Veluwe	17.67 [1.67:36.32]	-0.29 [-49.44:9.68]
Wytham Woods	Liesbos	9.54 [0.61:59.40]	0.06 [-65.72:19.80]
Wytham Woods	Oosterhout	24.95 [3.97:49.77]	-27.49 [-68.55:0.79]
Wytham Woods	Valencia	44.37 [14.83:87.95]	-71.65 [-118.90:3.45]
Hoge Veluwe	Liesbos	10.83 [0.98:56.96]	0.01 [-39.86:29.32]
Hoge Veluwe	Oosterhout	8.83 [0.22:36.58]	-0.10 [-31.73:23.61]
Hoge Veluwe	Valencia	77.34 [23.35:89.12]	-79.47 [-148.09:-6.86]
Liesbos	Oosterhout	8.70 [0.96:56.61]	-0.23 [-45.53:35.70]
Liesbos	Valencia	75.01 [19.86:89.79]	-79.16 [-139.23:31.36]
Oosterhout	Valencia	76.59 [25.60:89.96]	-97.43 [-151.98:0.89]

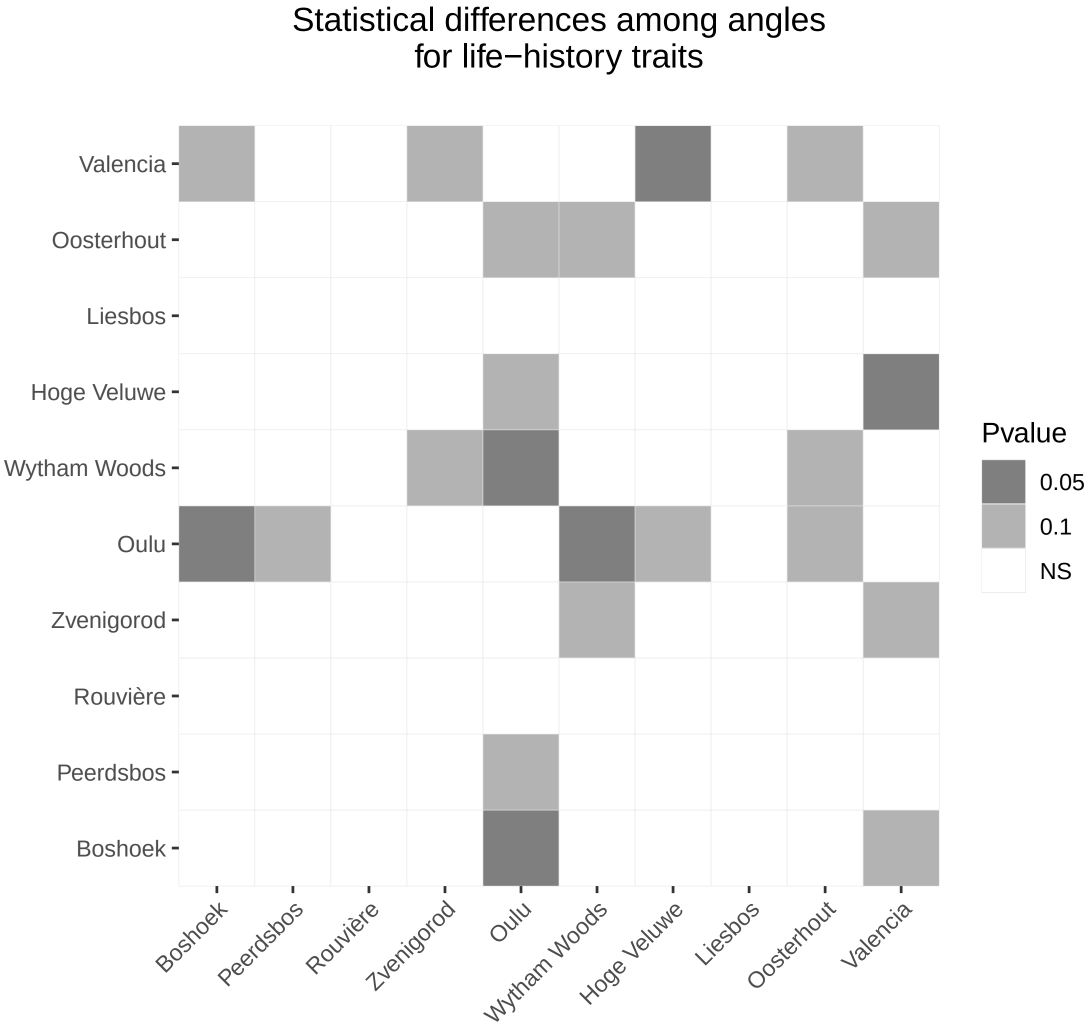

Figure S11: Statistical differences between g_{max} angles for the G-matrix of life-history traits. The statistical significant difference are provided at a respective p-value of 0.05 (dark grey) and 0.1 (light grey). Statistical support was estimated using the strategy described above.

Table S25: Angle values between g_{max} for each pair of populations for the G-matrix of morphological traits. Ψ are provided with the 95% Credible Interval (see above for details).

**Population 1**	**Population 2**	**Angle [95%CI]**	**Ψ [95%CI]**
Boshoek	Oulu	9.26 [1.08:35.22]	-0.05 [-25.56:15.74]
Boshoek	Peerdbos	11.19 [1.72:18.90]	-6.02 [-21.06:5.94]
Boshoek	Rouvière	16.59 [2.60:53.61]	-0.12 [-51.36:15.09]
Boshoek	Zvenigorod	16.19 [3.28:26.84]	-6.75 [-29.79:7.18]
Boshoek	Hoge Veluwe	12.52 [2.35:32.84]	-0.89 [-36.17:10.87]
Boshoek	Liesbos	8.03 [0.27:38.29]	-0.12 [-24.83:15.38]
Boshoek	Oosterhout	28.31 [7.93:46.30]	-30.95 [-61.24:0.27]
Boshoek	Valencia	9.23 [0.90:18.78]	-0.16 [-18.67:11.81]
Oulu	Peerdbos	15.68 [2.32:38.50]	-12.42 [-42.07:8.51]
Oulu	Rouvière	18.90 [0.65:59.97]	0.00 [-42.81:34.77]
Oulu	Zvenigorod	14.07 [0.52:39.83]	-0.93 [-32.00:19.50]
Oulu	Hoge Veluwe	7.34 [0.75:42.69]	-0.06 [-37.70:22.23]
Oulu	Liesbos	10.73 [0.87:52.35]	0.10 [-31.52:32.59]
Oulu	Oosterhout	24.14 [2.33:53.91]	-1.01 [-60.85:16.01]
Oulu	Valencia	7.41 [0.24:34.18]	0.10 [-26.22:21.90]
Peerdbos	Rouvière	27.27 [3.32:61.21]	-24.33 [-66.73:13.76]
Peerdbos	Zvenigorod	15.78 [5.11:32.74]	-20.93 [-39.73:2.45]
Peerdbos	Hoge Veluwe	18.23 [6.53:41.46]	-23.95 [-49.63:0.75]
Peerdbos	Liesbos	9.59 [0.93:38.89]	-0.11 [-29.70:12.87]
Peerdbos	Oosterhout	30.69 [17.36:56.77]	-46.56 [-81.86:-15.46]
Peerdbos	Valencia	17.78 [3.95:27.13]	-17.63 [-35.11:2.32]
Rouvière	Zvenigorod	18.47 [1.26:50.44]	-0.07 [-44.24:23.55]
Rouvière	Hoge Veluwe	15.31 [2.21:51.25]	-0.02 [-39.67:29.60]
Rouvière	Liesbos	21.91 [2.06:63.24]	-0.14 [-57.76:35.66]
Rouvière	Oosterhout	13.98 [0.90:49.26]	-0.85 [-39.08:28.91]
Rouvière	Valencia	21.90 [1.88:50.42]	-0.34 [-43.08:20.32]
Zvenigorod	Hoge Veluwe	9.23 [0.92:28.05]	-0.07 [-19.16:19.02]
Zvenigorod	Liesbos	18.30 [3.26:49.40]	-0.08 [-42.80:19.15]
Zvenigorod	Oosterhout	19.67 [3.55:41.92]	0.18 [-45.28:14.52]
Zvenigorod	Valencia	12.86 [1.22:26.40]	-1.35 [-27.95:13.14]
Hoge Veluwe	Liesbos	20.96 [2.21:48.18]	-0.98 [-49.80:20.74]
Hoge Veluwe	Oosterhout	12.98 [2.08:37.29]	-0.22 [-43.90:15.90]
Hoge Veluwe	Valencia	12.16 [1.74:31.24]	-3.85 [-37.50:12.48]
Liesbos	Oosterhout	31.64 [3.07:60.21]	-27.01 [-72.49:15.49]
Liesbos	Valencia	11.13 [0.56:41.97]	-0.10 [-27.80:23.13]
Oosterhout	Valencia	19.90 [2.90:41.21]	-29.58 [-52.32:7.25]

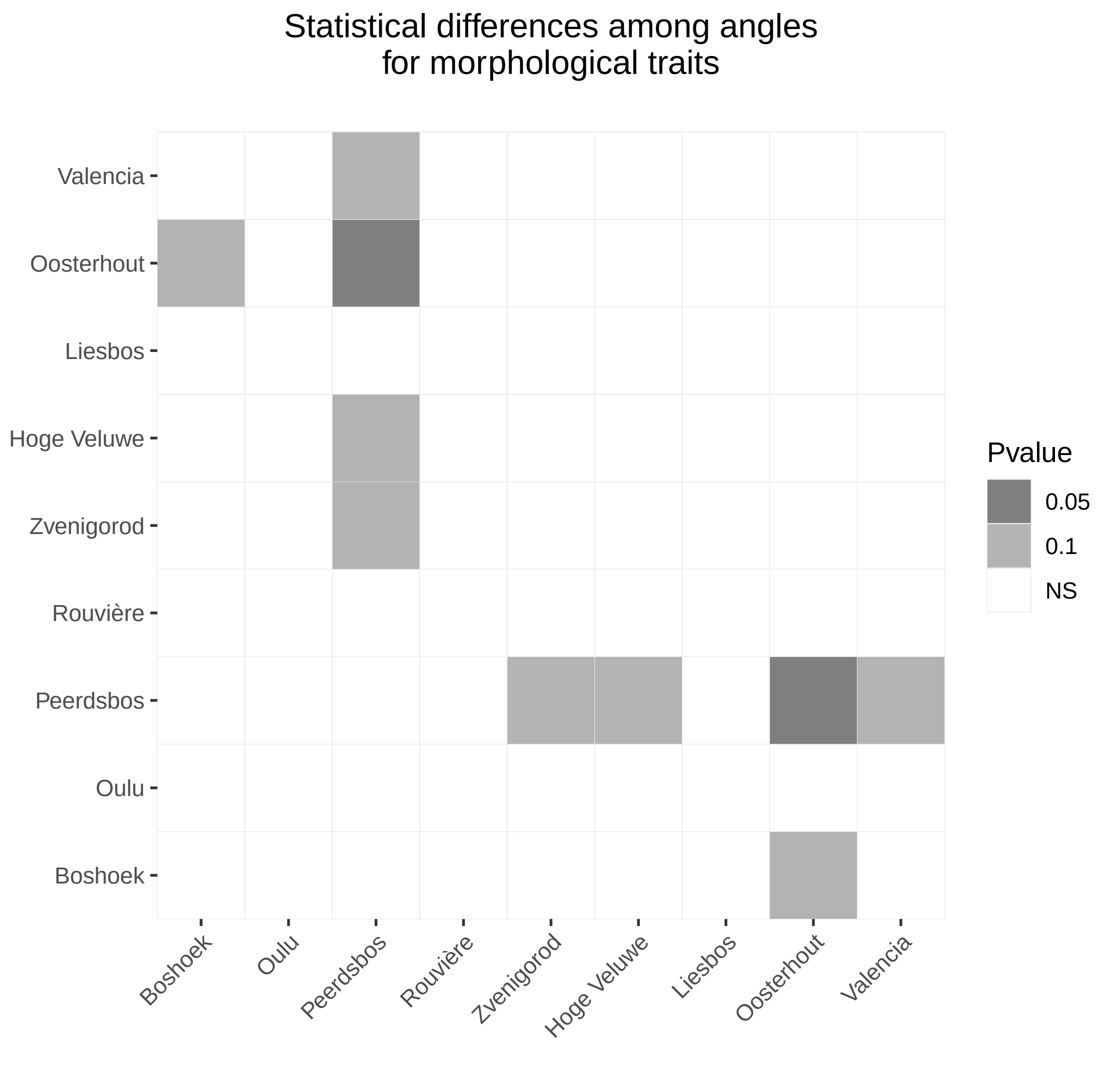

Figure S12: Statistical differences between g_{max} angles for the G-matrix of morphological traits. The statistical significant difference are provided at a respective p-value of 0.05 (dark grey) and 0.1 (light grey). Statistical support was estimated using the strategy described above.

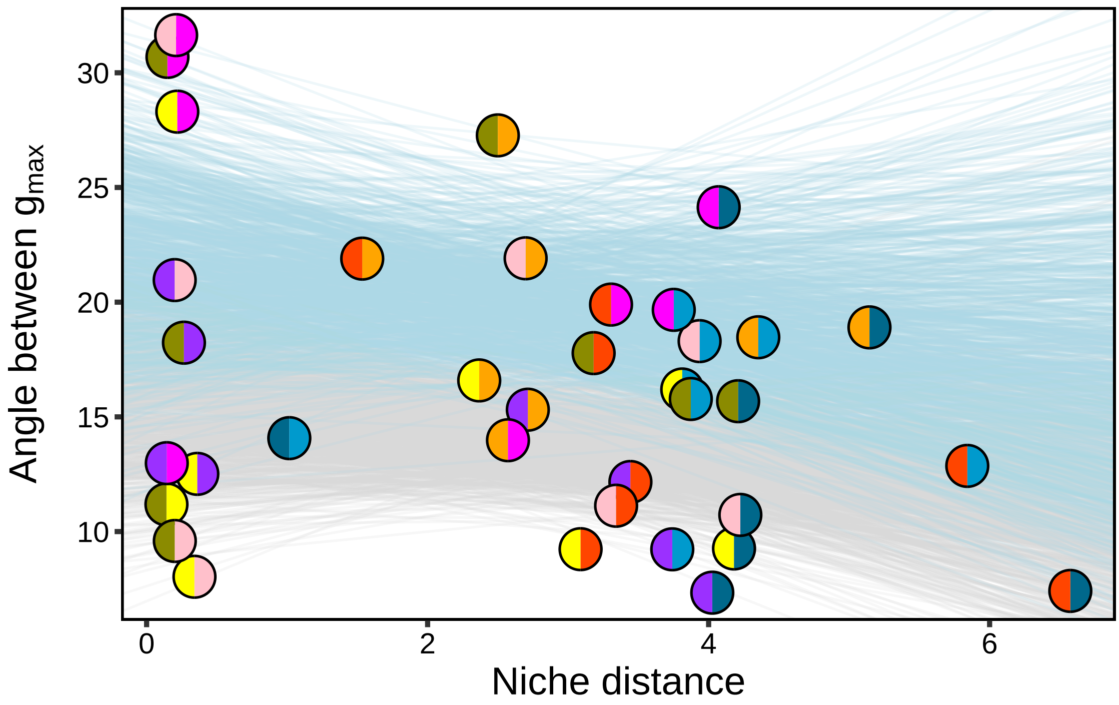

Figure S13: Difference in orientation between G-matrices of morphological traits. Relationship between the difference in g_{max} direction for each pair of populations and their respective niche distance. Coloured hemispheres refer to the population considered for pairwise comparison (see Fig.1 for details). The light blue and grey envelops represent the linear regressions realized on posterior distributions of distance estimated using data and null model respectively. Only regressions with a slope within 95% Credible Interval are shown.

Supporting Information 7: Representations of the G-matrices in the form of bidimensional ellipse, volume and eccentricity estimates

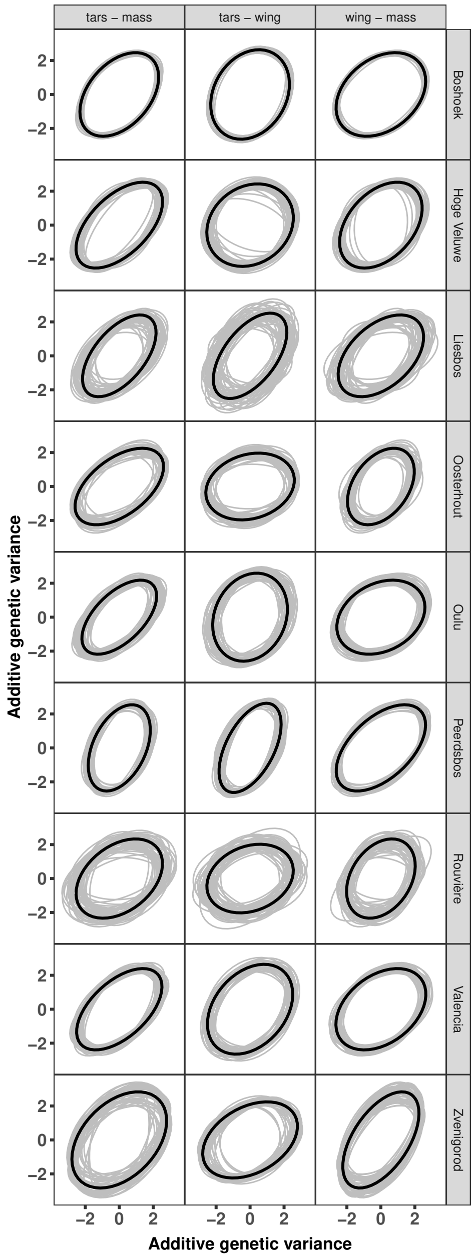

Figure S14: Standardized G-matrix in the form of bidimensional ellipse for morphological traits. The posterior modes are represented by black ellipses. The grey ellipses represent fifty random samples drawn from the posterior distribution. Standard data ellipses are represented, such that the half widths of their projections on axes x and y give the standard deviation of the corresponding traits. The x and y axes correspond to the first and second traits respectively mentioned in the upper panels.

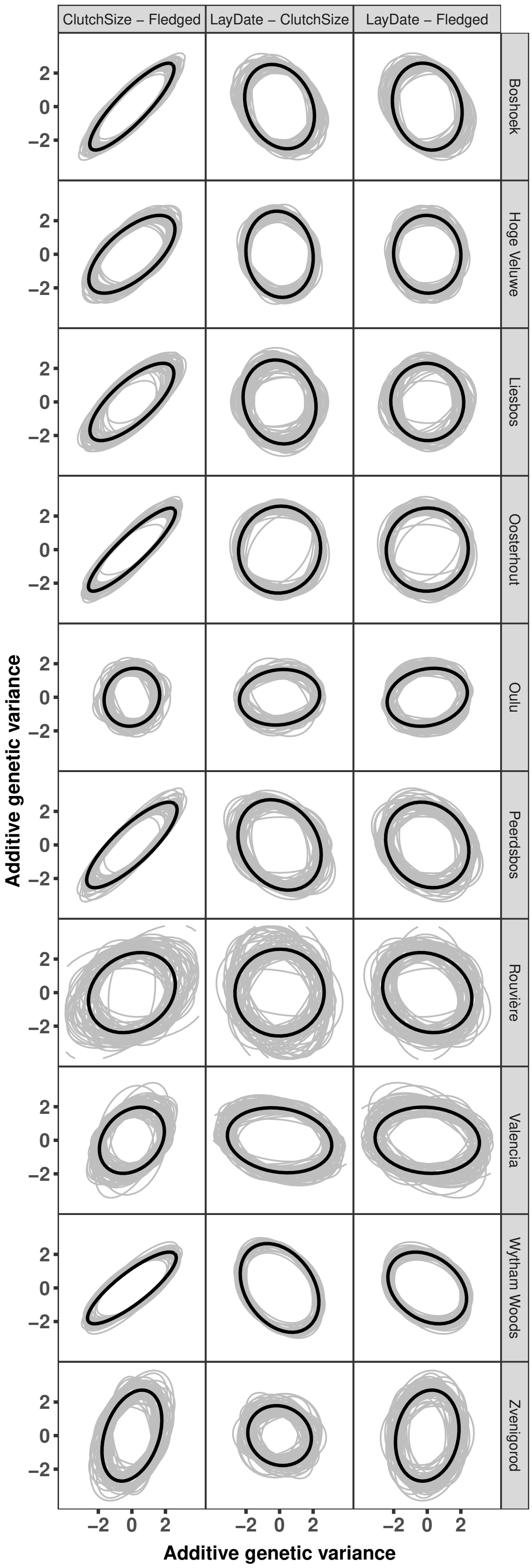

Figure S15: Standardized G-matrix in the form of bidimensional ellipse for life-history traits. The posterior modes are represented by black ellipses. The grey ellipses represent fifty random samples drawn from the posterior distribution. Standard data ellipses are represented, such that the half widths of their projections on axes x and y give the standard deviation of the corresponding traits. The x and y axes correspond to the first and second traits respectively mentioned in the upper panels.

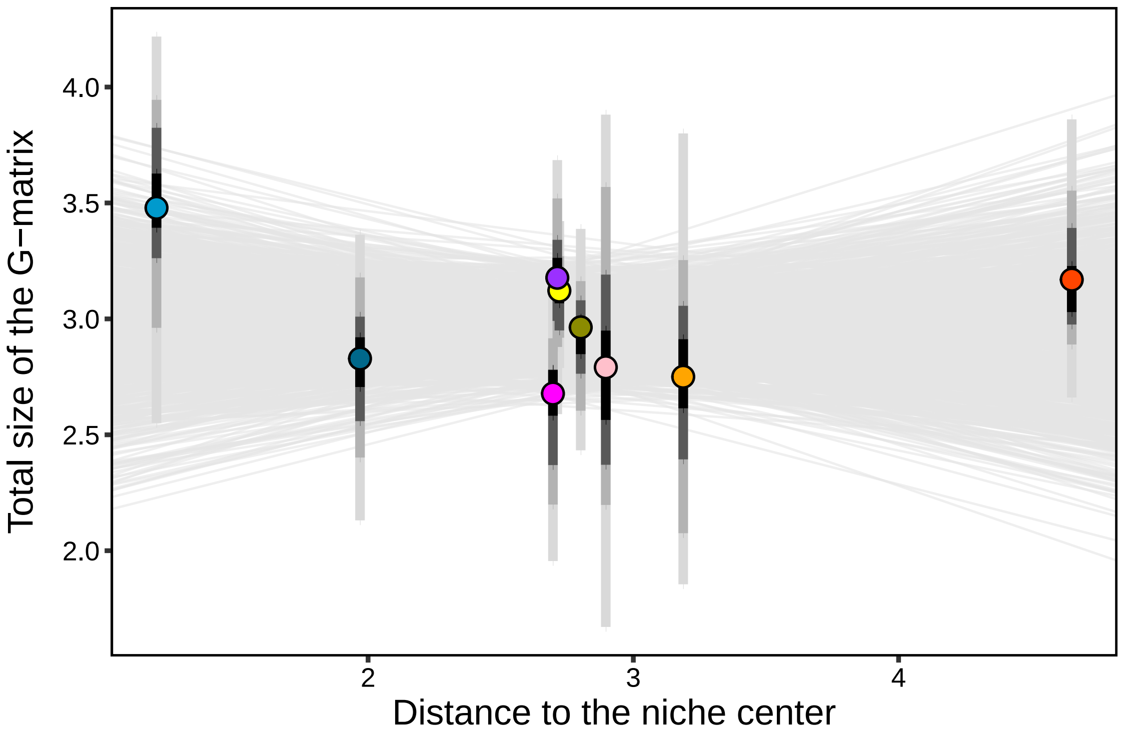

Figure S16: Total size of the G-matrices of morphological traits according to the distance of population to the niche center. Points represent the posterior mode estimates filled with colors corresponding to their respective populations and grey bars the 95%, 75% and 50% Credible Intervals (CI) (respectively light grey, grey and dark grey). The light grey envelop represents the linear regressions realized on posterior distributions (see materials and methods for details). The slope of linear regression was not significant at the 95% CI (slope[95%CI]=0.26[-0.13:0.93]). Only regressions with a slope within 95% CI are shown. Figure for life-history traits is provided in the main manuscript.

Table S26: Percent of variance explained by g_{max} for life-history and morphological G-matrix. Values correspond to posterior mode and brackets to the 95% Credible Interval.

**Population**	**% variance explained by g_{max} for life-history G-matrix**	**% variance explained by g_{max} for morphological G-matrix**
Oulu	0.55 [0.42:0.66]	0.57 [0.47:0.66]
Zvenigorod	0.62 [0.45:0.77]	0.63 [0.54:0.76]
Hoge Veluwe	0.63 [0.50:0.72]	0.60 [0.50:0.68]
Liesbos	0.63 [0.50:0.78]	0.64 [0.48:0.79]
Oosterhout	0.67 [0.54:0.75]	0.61 [0.50:0.71]
Boshoek	0.68 [0.59:0.81]	0.55 [0.51:0.60]
Peerdbos	0.67 [0.54:0.78]	0.68 [0.60:0.73]
Wytham Woods	0.69 [0.60:0.75]	**
Rouvière	0.55 [0.42:0.71]	0.59 [0.43:0.71]
Valencia	0.63 [0.47:0.75]	0.62 [0.53:0.69]

References

Aguirre, J. D., E. Hine, K. McGuigan, and M. W. Blows. 2013. Comparing G: multivariate analysis of genetic variation in multiple populations. Heredity (Edinb). 1–9.

Hansen, T. F., and D. Houle. 2008. Measuring and comparing evolvability and constraint in multivariate characters. J. Evol. Biol. 21:1201–19.

Hansen, T. F., C. Pélabon, and D. Houle. 2011. Heritability is not Evolvability. Evol. Biol. 38:258–277.

Hine, E., S. F. Chenoweth, H. D. Rundle, and M. W. Blows. 2009. Characterizing the evolution of genetic variance using genetic covariance tensors. Proc. R. Soc. B Biol. Sci. 364:1567–78.

Houle, D., C. Pélabon, G. P. Wagner, and T. F. Hansen. 2011. Measurement and Meaning in Biology. Q. Rev. Biol., doi: 10.1086/658408.

Kuznetsova, A., Brockhoff, P.B. & Christensen, R.H.B. (2017) lmerTest Package: Tests in Linear Mixed Effects Models. Journal of Statistical Software, 82, 1–26.

Robinson, M.R. & Beckerman, A.P. (2013) Quantifying multivariate plasticity: genetic variation in resource acquisition drives plasticity in resource allocation to components of life history. Ecology letters, 16, 281–90.
